# Data-driven identification of ageing-related diseases from electronic health records

**DOI:** 10.1038/s41598-021-82459-y

**Published:** 2021-02-03

**Authors:** Valerie Kuan, Helen C. Fraser, Melanie Hingorani, Spiros Denaxas, Arturo Gonzalez-Izquierdo, Kenan Direk, Dorothea Nitsch, Rohini Mathur, Constantinos A. Parisinos, R. Thomas Lumbers, Reecha Sofat, Ian C. K. Wong, Juan P. Casas, Janet M. Thornton, Harry Hemingway, Linda Partridge, Aroon D. Hingorani

**Affiliations:** 1 Institute of Health Informatics, University College London, London, UK; 2 Health Data Research UK London, University College London, London, UK; 3 University College London British Heart Foundation Research Accelerator, London, UK; 4 Institute of Healthy Ageing, Department of Genetics, Evolution and Environment, University College London, London, UK; 5 Moorfields Eye Hospital, London, UK; 6 Alan Turing Institute, London, UK; 7 Department of Non-communicable Disease Epidemiology, London School of Hygiene and Tropical Medicine, London, UK; 8 Barts Heart Centre, St Bartholomew’s Hospital, London, UK; 9 School of Pharmacy, University College London, London, WC1N 1AX UK; 10 Centre for Safe Medication Practice and Research, Department of Pharmacology and Pharmacy, The University of Hong Kong, Pok Fu Lam, Hong Kong; 11 Department of Medicine, Brigham and Women’s Hospital, Harvard Medical School, Boston, MA USA; 12 Massachusetts Veterans Epidemiology Research and Information Center (MAVERIC), VA Boston Healthcare System, Boston, MA USA; 13 European Molecular Biology Laboratory - European Bioinformatics Institute EMBL-EBI, Wellcome Genome Campus, Hinxton, Cambridgeshire, CB10 1SD UK; 14 The National Institute for Health Research University College London Hospitals Biomedical Research Centre, University College London, London, W1T 7DN UK; 15 Max Planck Institute for Biology of Ageing, Cologne, Germany; 16 Institute of Cardiovascular Science, University College London, London, UK

**Keywords:** Cancer, Cardiovascular diseases, Endocrine system and metabolic diseases, Eye diseases, Gastrointestinal diseases, Haematological diseases, Immunological disorders, Infectious diseases, Kidney diseases, Metabolic disorders, Neurological disorders, Nutrition disorders, Psychiatric disorders, Reproductive disorders, Respiratory tract diseases, Rheumatic diseases, Skin diseases, Urogenital diseases, Geriatrics, Public health, Epidemiology

## Abstract

Reducing the burden of late-life morbidity requires an understanding of the mechanisms of ageing-related diseases (ARDs), defined as diseases that accumulate with increasing age. This has been hampered by the lack of formal criteria to identify ARDs. Here, we present a framework to identify ARDs using two complementary methods consisting of unsupervised machine learning and actuarial techniques, which we applied to electronic health records (EHRs) from 3,009,048 individuals in England using primary care data from the Clinical Practice Research Datalink (CPRD) linked to the Hospital Episode Statistics admitted patient care dataset between 1 April 2010 and 31 March 2015 (mean age 49.7 years (s.d. 18.6), 51% female, 70% white ethnicity). We grouped 278 high-burden diseases into nine main clusters according to their patterns of disease onset, using a hierarchical agglomerative clustering algorithm. Four of these clusters, encompassing 207 diseases spanning diverse organ systems and clinical specialties, had rates of disease onset that clearly increased with chronological age. However, the ages of onset for these four clusters were strikingly different, with median age of onset 82 years (IQR 82–83) for Cluster 1, 77 years (IQR 75–77) for Cluster 2, 69 years (IQR 66–71) for Cluster 3 and 57 years (IQR 54–59) for Cluster 4. Fitting to ageing-related actuarial models confirmed that the vast majority of these 207 diseases had a high probability of being ageing-related. Cardiovascular diseases and cancers were highly represented, while benign neoplastic, skin and psychiatric conditions were largely absent from the four ageing-related clusters. Our framework identifies and clusters ARDs and can form the basis for fundamental and translational research into ageing pathways.

## Introduction

Genetic association studies and experimental trials in humans and animal models have revealed that mechanisms of ageing contribute to the aetiology of the diseases of older age^1–5^. Processes such as DNA damage, mitochondrial and stem cell dysfunction, impaired proteostasis and cellular senescence are each present in the aetiology of multiple diseases, potentially contributing to overlapping aetiology^2,4^.

A deeper understanding of the shared and distinct mechanisms leading to the diseases of ageing requires empirical specification of which diseases are ageing-related. Furthermore, an accurate classification of disease onset could identify clusters of diseases with common contributions from the ageing process. A starting point should include a framework for identifying diseases that become more common in the older members of the population, and an approach for detecting different patterns of disease incidence with increasing age.

The majority of studies on ageing refer to “age-related” or “ageing-related” diseases without specifying how the terms were derived, nor how such diseases were identified^6–10^. One study measuring population ageing using the Global Burden of Disease Study 2017 defined “age-related diseases” as those with incidence rates among the adult population that increased quadratically with age^10^. This study did not use directly measured incidence data, but was based on estimates derived from a statistical model. Another study used medical claims data from a Brazilian insurance company to cluster age density patterns of raw ICD-10 codes but did not specifically identify diseases that increased with age.

Large-scale, population-based EHRs from universal cradle-to-grave health systems provide the optimal setting to measure and discover patterns of disease incidence with age. In order to capture the population experience of age-related diseases, we analysed the relationship of 289 diseases that involve intensive use of health-care resources, using aggregated data from Electronic Health Records (EHRs) for 3,009,048 individuals in a large, representative-population dataset in England between 1 April 2010 and 31 March 2015^11^.

We propose a standard terminology and methodology to define diseases that increase in frequency with age. We use a standardised term—“ageing-related diseases” (ARDs)—to refer to diseases that accumulate with increasing age, and the term “age-related” to refer to diseases that occur within specific age ranges^12,13^. We used two complementary approaches to distinguish diseases of ageing from diseases for which increasing age is not a risk factor. First, we applied cluster analysis in order to group diseases with similar disease onset patterns with respect to age. This identified nine main disease clusters, four of which consisted of diseases that increased in incidence with age, although with strikingly different age-related patterns, suggestive of differing aetiologies. Second, we assessed how well the observed age-specific disease onset rates from the EHR data corresponded to actuarial models in order to determine the likelihood that a disease was ageing-related.

## Methods

### Dataset

We used the Clinical Practice Research Datalink (CPRD), a large, clinically representative primary care database linked to the Hospital Episode Statistics admitted patient care (HES-APC) dataset in England that has previously been validated for epidemiological research^14^. Individuals were included in the study if they had been registered for at least a year in a participating general practice between 1 April 2010 and 31 March 2015, were aged above 20 years during this period, and their individual and practice records met research standards set by the CPRD.

The study was approved by the Independent Scientific Advisory Committee for the Medicines and Healthcare products Regulatory Agency (protocol 16_022). CPRD has ethics approval from the Health Research Authority to support research using anonymised patient data. Primary care practices provide consent for CPRD to collect de-identified primary care data from their practice. Individual patients can opt-out of sharing their data for research and CPRD does not collect data for these patients. Therefore, informed consent is given at the time of data collection and does not need to be repeated for each study. We confirm that data were analysed in accordance with the relevant guidelines and regulations.

### Disease selection

The selection process for diseases was based on the number of Hospital Episode Statistics (HES) finished consultant episodes (FCEs) (the time spent under the care of one consultant whilst admitted to hospital) in England, prevalence estimates and clinical importance as described in a previous study^11^. Briefly, diseases that had more than 10,000 FCEs were included. If a disease had fewer than 10,000 FCEs, it was included in the study if the prevalence was higher than 0.01% and it was considered to be clinically important by a panel of clinicians^11^. Phenotyping algorithms defining these diseases were based on clinical measurements recorded in CPRD, or diagnosis and procedural codes recorded in CPRD and HES. These algorithms are available on the CALIBER platform (https://portal.caliberresearch.org and https://github.com/spiros/chronological-map-phenotypes)^11,15,16^. After excluding pregnancy-related conditions, symptoms, signs, abnormal clinical and laboratory findings, external causes of morbidity and mortality, congenital diseases and perinatal conditions, 289 diseases were analysed in this study. Diseases were organised into 15 categories corresponding closely to International Classification of Diseases, tenth revision (ICD-10) chapters (Supplementary Table S1).

### Age of disease onset

The age of disease onset was approximated by the age at which an individual was first recorded with a specific condition. The age at first reported diagnosis was the earliest age at which the criteria in a phenotyping algorithm for a specific condition were met from any source in the EHRs prior to 31 March 2015. In order to exclude diseases that may have occurred as a result of developmental processes from childhood through to puberty, we omitted diagnoses for ages 20 years and lower, in line with the WHO’s definition of adolescence as the period between 10 and 19 years of age^17,18^. We also excluded new diagnoses made after the age of 85 years because of the low onset of previously undiagnosed disease above this age. Individuals alive beyond this age may be subject to survival bias, representing an unusually robust subset of the population who are less susceptible to ARDs^19^.

### Rate of disease onset

The rate of disease onset was represented by the rate at which the first reported diagnosis appeared in the pooled electronic health records (EHR). The terms “rate of disease onset”, “rate of disease diagnosis” and “rate of first recorded diagnosis” are used interchangeably in this report.

For integer year of age *x* = 21,…, 84, we calculated *q*_*x*_, the age-specific rate of disease onset for each disease:

1 $$q_{x} = d_{x} /l_{x} ,$$

where, *d*_*x*_ = number of patients first recorded with the disease at age *x*, *l*_*x*_ = number of patients with no record for the disease at age *x.*

### Clustering the age-specific rate of disease onset curves

The rate of first recorded diagnosis was plotted against age to summarise an age-specific disease onset curve between 20 to 85 years for each of the 289 conditions studied (Supplementary Figs. S1–S10). For each disease, the rate of disease onset at each year of age was standardised by dividing it by the sum of the age-specific rates of disease onset from age *x* = 21,…, 84:

2 $${\text{Standardised}} \; \text{age-specific} \; \text{rate} \; \text{of} \; \text{disease} \; \text{onset}: {{\widehat q}_x}=\frac{{q}_{x}}{\sum_{i=21}^{84}{q}_{i}}$$

Euclidean distances between the standardised rates of disease onset for every disease pair were calculated at each year of age. We explored four different clustering techniques for the age-specific rate of disease onset curves: hierarchical agglomerative clustering; k-means clustering; k-medioid clustering (partitioning around medioids (PAM)); and spectral clustering^20–22^.

In hierarchical clustering, the dissimilarity between two clusters can be measured using different linkage methods. Using the cophenetic correlation coefficient, we determined that the average linkage method was optimal for hierarchical clustering of the age-specific rate of disease onset curves (see Supplementary Notes, Supplementary Table S2). The optimal number of clusters for each of the four clustering algorithms we explored was ascertained using the gap statistic proposed by Tibshirani et al.^23^. These were: 18 clusters for hierarchical agglomerative clustering with average linkage, 9 for k-means, 18 for PAM and 10 for spectral clustering (see Supplementary Notes, Supplementary Table S3). Finally, we used the Dunn validation index^24^ to select the optimal clustering algorithm out of the four that we tested (see Supplementary Notes, Supplementary Table S3). The hierarchical agglomerative clustering algorithm with average linkage and 18 clusters had the highest Dunn value. The results from this algorithm are reported in this article. The 18 clusters were separated into nine “main” clusters with three or more diseases in each cluster, and nine “outlier” clusters containing just one or two diseases.

### Modelling the relationship between rate of disease onset and age

ARDs should, by definition, have rates of disease onset that increase with age. Physiological decline with advancing age, or senescence, is manifested in populations as an increase in mortality rate at older ages. This physiological decline is caused by ageing processes that lead to diseases that result in death^25^. Therefore, assuming that the distribution function of disease onset for ARDs resembles that for mortality, we applied the Gompertz function (an actuarial model that was originally designed to describe human mortality)^26^:

3 $$q_{x} = \, \alpha e^{\beta x}$$

where, *q*_*x*_ = age-specific rate of disease onset at age *x* (from Eq. (1)), *α* = baseline rate of disease onset at age *x* = 21, *β* = senescent (age-dependent) component (rate of disease onset increase over age).

Under the Gompertz model, log (*q*_*x*_) is a linear function of age *x*:

4 $${\text{log }}\left( {q_{x} } \right) \, = {\text{ log}}\alpha + \beta x$$

If *β*, the coefficient of the age variable in the Gompertz model is negative, the curve is downward sloping, and hence the rate of disease onset decreases with age, indicating that the disease is not ageing-related.

Some diseases may not have rates of onset that increase monotonically with age, but could still be considered ageing-related. Examples include diseases with one or more small local peaks earlier in life followed by a much greater increase with advancing age, or those with an exponential increase preceding a subsequent decline or levelling off in later life. In these circumstances, an exponential-polynomial model, such as the Gompertz–Makeham (GM) model^27^, may fit the data better:

5 $$q_{x} = {\text{ exp}}\left\{ {{\text{pol}}\left( x \right)} \right\}$$

Here we define pol(*x*) as a quadratic term such that

6 $${\text{log }}\left( {q_{x} } \right) = {\text{ log}}a + bx + \, cx^{2}$$

### Goodness-of-fit of the Gompertz–Makeham model

The R-squared (R^2^), a statistical measure of how close the data are to the fitted regression line, is often used to assess how well a model fits the data. In this study, it is the proportion of variation in the independent age variable that is explained by the model. The R-squared increases with the addition of each new independent variable to the model. Therefore, the polynomial Gompertz–Makeham model with the additional quadratic age term will always have a higher R^2^ than the Gompertz model. The adjusted R^2^ is a modification of the R^2^ that increases only if an additional variable improves the model more than would be expected by chance and decreases when the improvement is less than expected by chance. We used the adjusted R^2^ to determine whether the Gompertz–Makeham model was a good fit for the observed epidemiological data.

### Algorithm for assigning the likelihood that a disease is ageing-related

**Figure 1:**
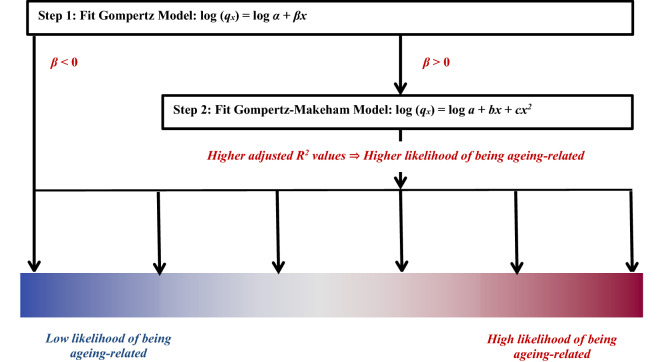
Algorithm for determining the likelihood that a disease is ageing-related. This depends on β, the age coefficient of the Gompertz model and the adjusted R^2^ of the Gompertz–Makeham model for each disease. *q*_*x*_ is the age-specific rate of disease onset at age *x*. *α, β, a, b,* and *c* are constants.

The following step-wise algorithm was applied to determine the likelihood that a condition was ageing-related (Fig. 1):

1. First, the Gompertz model (Eq. (4)) was fitted to the empirical data. If *β*, the coefficient of the age variable in the Gompertz model was negative, indicating that the disease onset decreased with increasing age, the condition was considered to have a very low likelihood of being ageing-related.
2. Next, the Gompertz–Makeham (GM) model (Eq. (6)) was fitted to the empirical data. Higher values of the adjusted R^2^ of the GM model were deemed to have higher likelihoods of being ageing-related.

We demonstrated which diseases were more likely to be ageing-related based on different bands of adjusted R^2^ with thresholds of 0.95, 0.90, 0.85 and 0.80 so that readers can observe the likelihood that a disease is ageing-related across a gradient.

All analyses were performed using R 3.5.0.

## Results

### Sample characteristics

We studied 3,009,048 individuals in a large, representative, population dataset in England between 1 April 2010 and 31 March 2015. The mean age was 49.7 years (standard deviation 18.6 years), 51% were female, and 70% were of white ethnicity. The median follow-up was 3.7 years (IQR: 1.5–5.0 years). The number of cases and median (interquartile range (IQR)) age of first recorded diagnosis above 20 years for 289 diseases is reported in Supplementary Table S1.

### Disease clusters defined by age-specific onset

**Figure 2:**
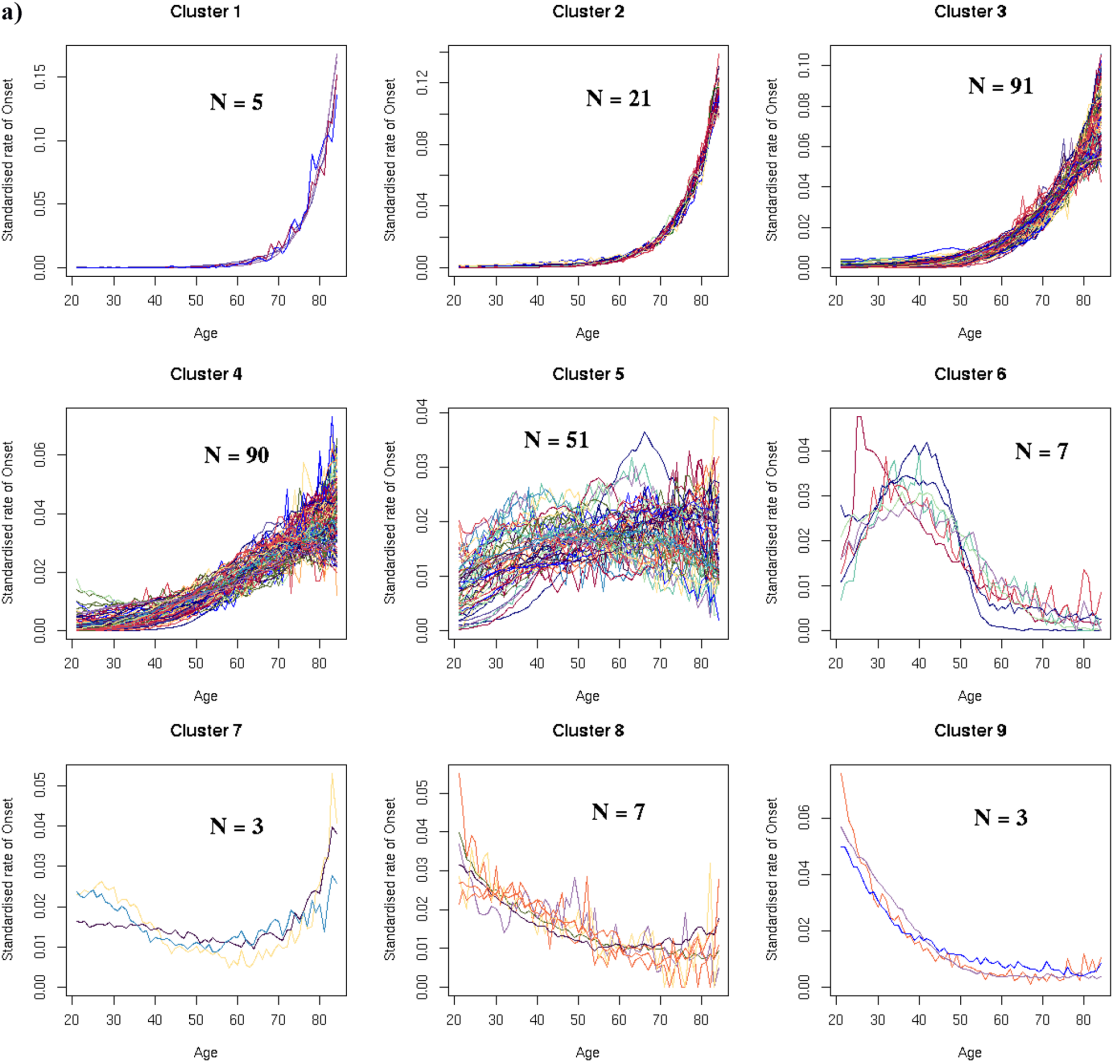

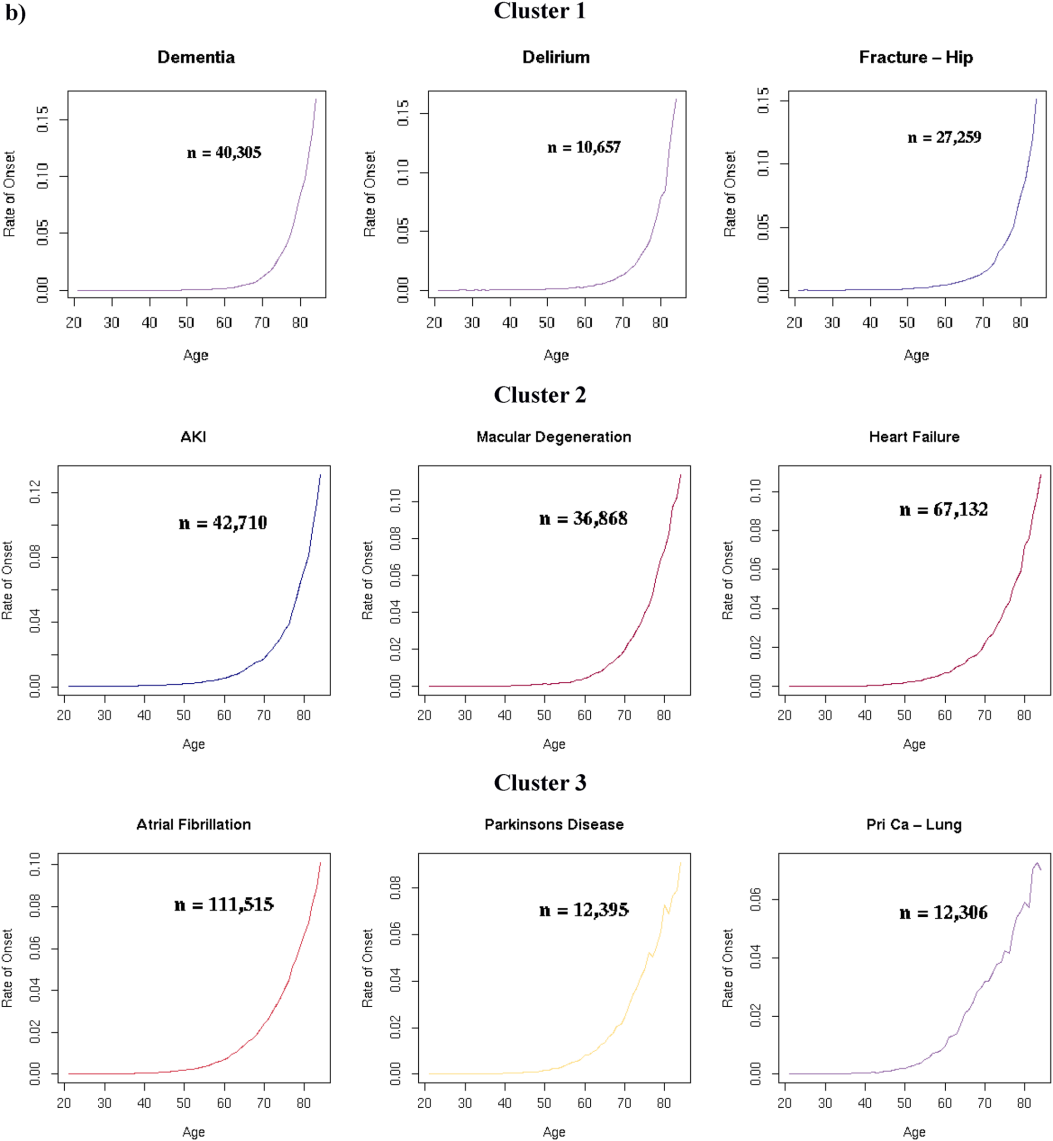

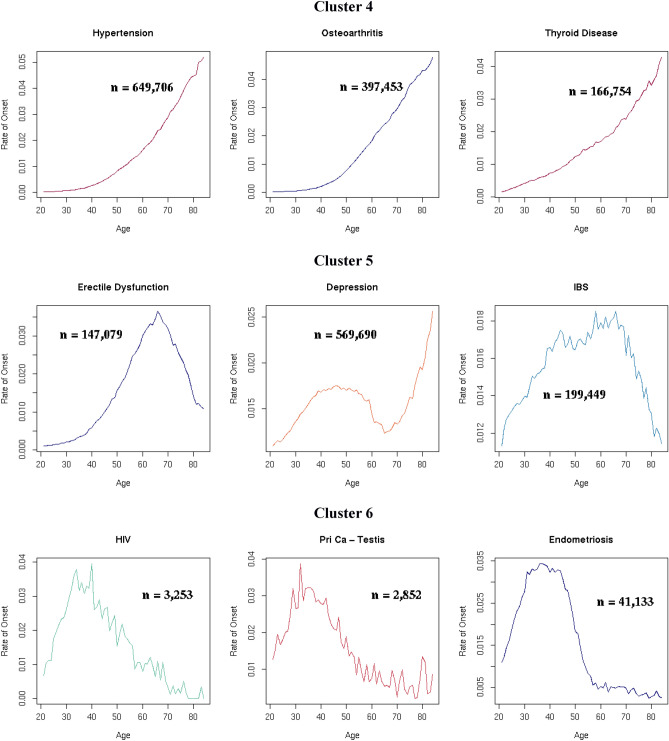

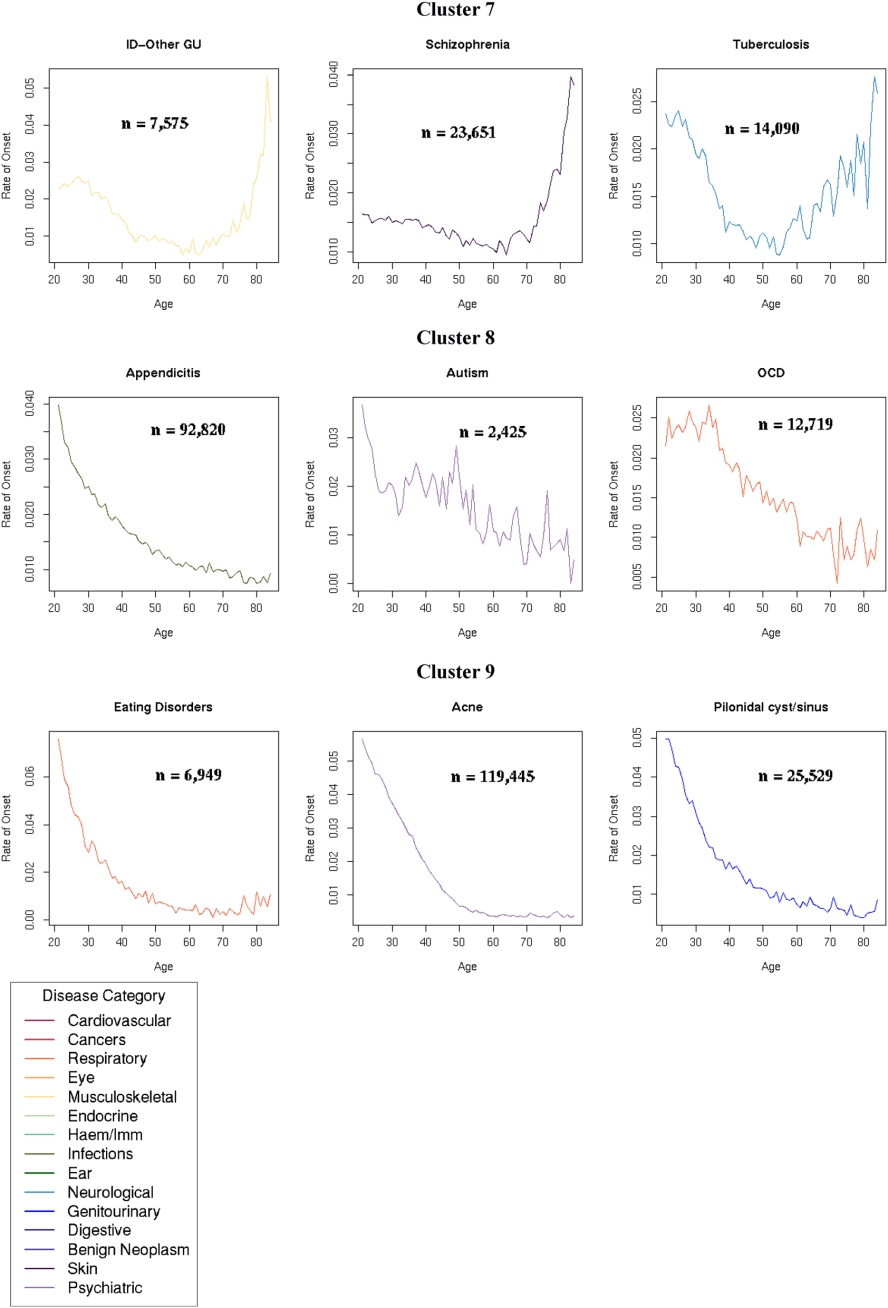
(**a**) In a data-driven approach, hierarchical clustering techniques were used to derive nine clusters of standardised age-specific rate of disease onset curves. The y-axis scales differ for each cluster. N (number of conditions in each cluster) is indicated in each cluster plot. (**b**) Age-specific rate of onset curves (not standardised) for examples from each cluster. The y-axis scales differ for each disease. The number of individuals between the ages of 20 and 85 years with the disease (n) is indicated in each plot.

Nine main clusters of disease onset patterns consisting of three or more diseases were identified for 278 diseases using a hierarchical, agglomerative clustering algorithm applied to standardised rate of disease onset curves for 289 diseases (Fig. 2a). This algorithm was selected following an evaluation of four different clustering methods using a set of objective criteria. The remaining eleven diseases fell into nine outlier clusters with two or fewer diseases each (Supplementary Table S1, Supplementary Fig. S1). Diseases that exemplify the different main clusters are shown in Fig. 2b. Supplementary Table S1 lists the main and outlier clusters to which each of the 289 diseases was assigned. Supplementary Figs. S2–S10 illustrate the age-specific rates of onset for each disease in the nine main clusters.

Diseases in Clusters 1, 2, 3 and 4 increased in incidence with age. The 5 diseases in Cluster 1 and 21 diseases in Cluster 2 had low age-specific rates of disease onset early in life followed by exponential growth at later ages, with a steeper rate of increase in Cluster 1 starting at a later age. Cluster 3 had 91 diseases that also showed exponential growth, but with disease onset rates that increased at an earlier age than in Clusters 1 and 2. Cluster 4 contained 90 diseases with rising rates of disease onset, but the increase was more linear and gradual, and started earlier than in Clusters 1, 2 and 3.

The relationship between age and disease onset in Cluster 5 was less clear. Most of the 51 curves in this heterogeneous cluster showed a small initial increase in rates of onset from the age of 20 years which levelled out or began to decline around the age of 40 years or later.

All seven diseases in Cluster 6 declined in onset between the ages of 20 to 50 years. Cluster 7 consisted of three diseases with relatively high rates of disease onset in young adulthood that declined steadily till the age of 60 years before increasing again. The seven diseases in Cluster 8 and three diseases in Cluster 9 all declined with age. The rate of decline in Cluster 9 was sharper than in Cluster 8.

**Figure 3:**
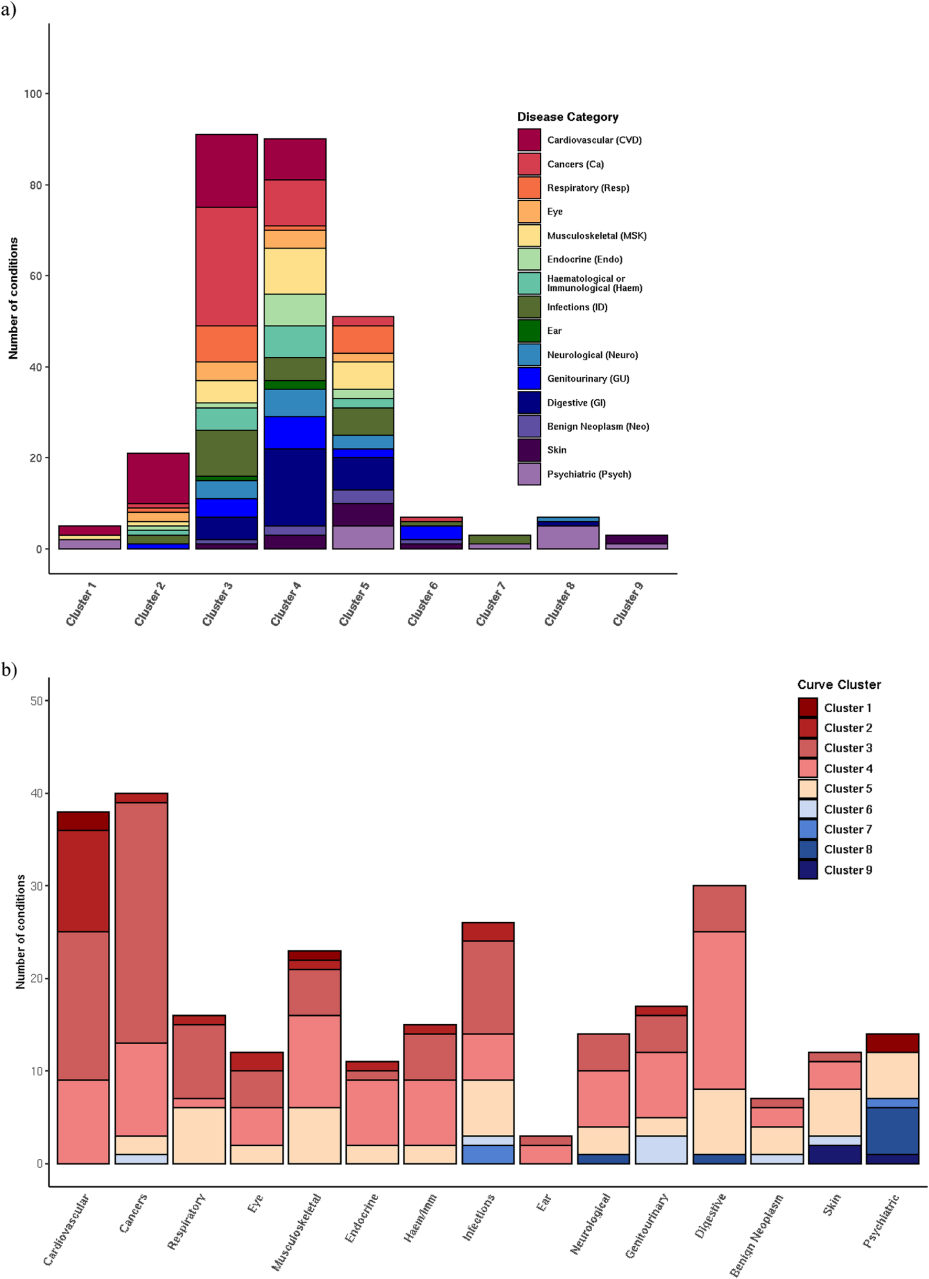
The relationship between disease category and age curve cluster for 278 diseases: (**a**) Diseases in each age cluster by disease category. (**b**) Diseases in each disease category by age curve cluster. The number of diseases in each disease category and age curve cluster is shown in Table 1.

Clusters 1, 2 and 3 were the most strongly associated with ageing. Cluster 1 comprised dementia, delirium, cardiac conduction deficits including trifascicular block and bifascicular block, as well as hip fracture. Cardiovascular diseases (CVDs) made up the highest proportion of the diseases in Cluster 2, and cancers the highest proportion in Cluster 3. Cluster 4 was also associated with ageing, with digestive system diseases comprising the largest category. Diseases spanning a wide range of disease categories were represented in these four clusters (Fig. 3a, Table 1). All CVDs studied, all ear diseases, and 37 out of 41 cancers were in Cluster 1, 2, 3 or 4. The three disease categories with the lowest proportion of diseases in Clusters 1 to 4 were benign neoplastic, skin and psychiatric diseases (Fig. 3b, Table 1).

**Table 1:** The relationship between disease category and age curve cluster for 278 diseases. The number of diseases is shown for each age curve cluster and disease category.

	Cluster 1	Cluster 2	Cluster 3	Cluster 4	Cluster 5	Cluster 6	Cluster 7	Cluster 8	Cluster 9	Total
Cardiovascular	2	11	16	9						38
Cancers		1	26	10	2	1				40
Respiratory		1	8	1	6					16
Eye		2	4	4	2					12
Musculoskeletal	1	1	5	10	6					23
Endocrine		1	1	7	2					11
Haematological or Immunological		1	5	7	2					15
Infections		2	10	5	6	1	2			25
Ear			1	2						3
Neurological			4	6	3			1		14
Genitourinary		1	4	7	2	3				17
Digestive			5	17	7			1		30
Benign neoplasms			1	2	3	1				7
Skin			1	3	5	1			2	12
Psychiatric	2				5		1	5	1	14
Total	5	21	91	90	51	7	3	7	3	278

### Median age of first recorded diagnosis

**Figure 4:**
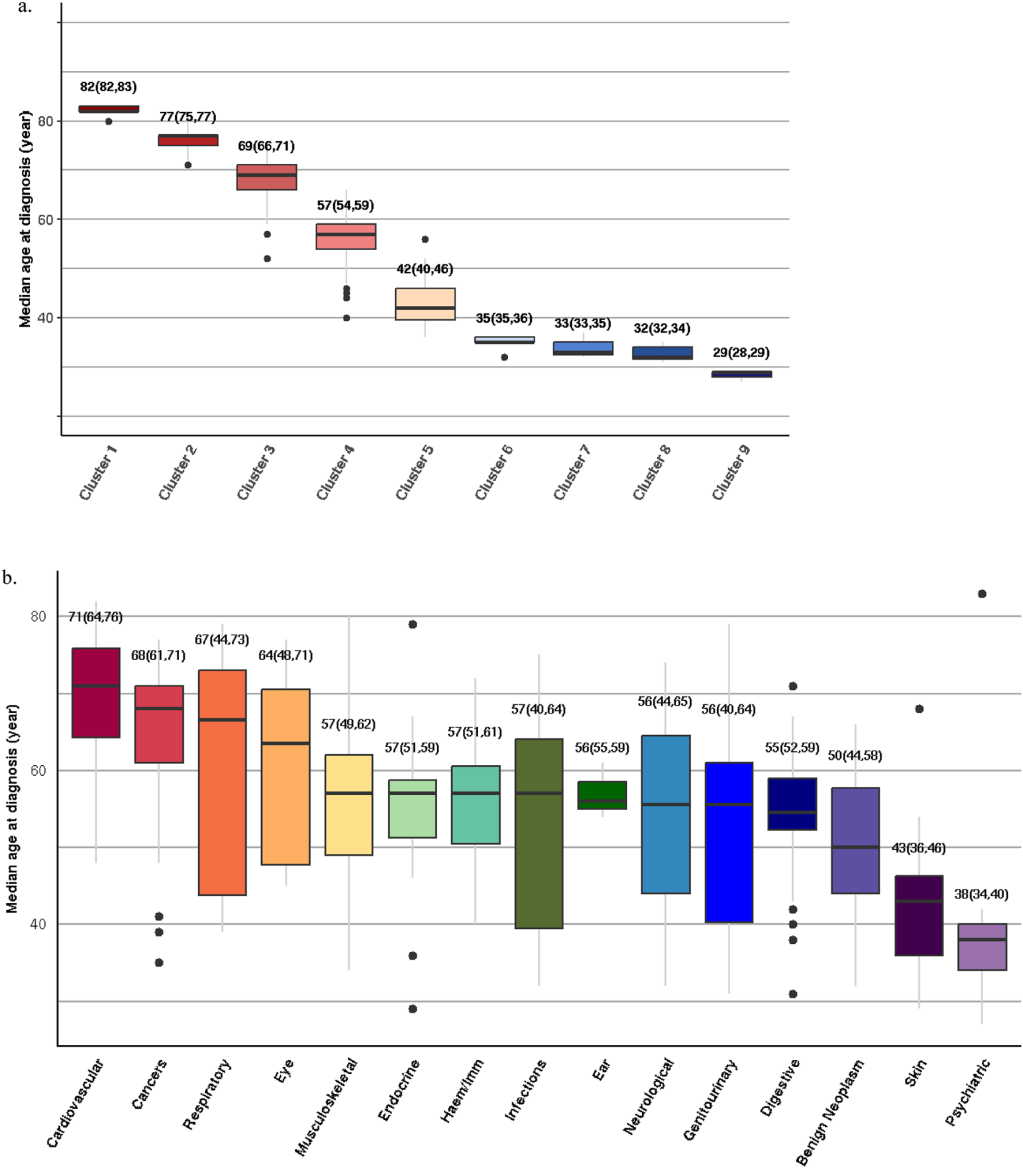
Median age of onset for 278 diseases in each curve cluster and disease category: (**a**) Box and whisker plots of the median age of first recorded diagnosis above the age of 20 years for diseases in each curve cluster; (**b**) Box and whisker plots of the median age of first recorded diagnosis (above the age of 20 years) for the 289 conditions grouped into 15 disease categories. The horizontal line inside the boxes represents the median, the upper and lower edges of the boxes represent the 25th and 75th percentiles, and the end-points of the upper and lower whiskers represent the highest and lowest values within 1.5*IQR, where IQR is the interquartile range. Numbers above the boxes indicate the median (25th percentile, 75th percentile).

The median age of first recorded diagnosis above the age of 20 years was highest for diseases in Cluster 1 (82y (82–83)), followed by those in Cluster 2 (77y (75–77)), Cluster 3 (69y (66–71)), Cluster 4 (57y (54–59)), Cluster 5 (42y (39.5–46)), Cluster 6 (35y (35–36)), Cluster 7 (33y (32.5–35)), Cluster 8 (32y (31.5–34)), and Cluster 9 (29y (28–29)) (Fig. 4a).

CVDs had the highest median age of diagnosis above the age of 20 years (median age, (interquartile range (IQR)): 71y (64–76)), followed by malignant (68y (61–71)), respiratory (67y (44–73)), eye (64y (48–71)), musculoskeletal (57y (49–62)), endocrine (57y (51–59)), haematological or immunological (57y (51–61)), infectious (57y (40–64)), ear (56y (55–59)), neurological (56y (44–65)), genitourinary (56y (40–64)), digestive (55y (52–59)), benign neoplastic (50y (44–58)), skin (43y (36–46)), and psychiatric (38y (34–40)) diseases (Fig. 4b).

**Figure 5:**
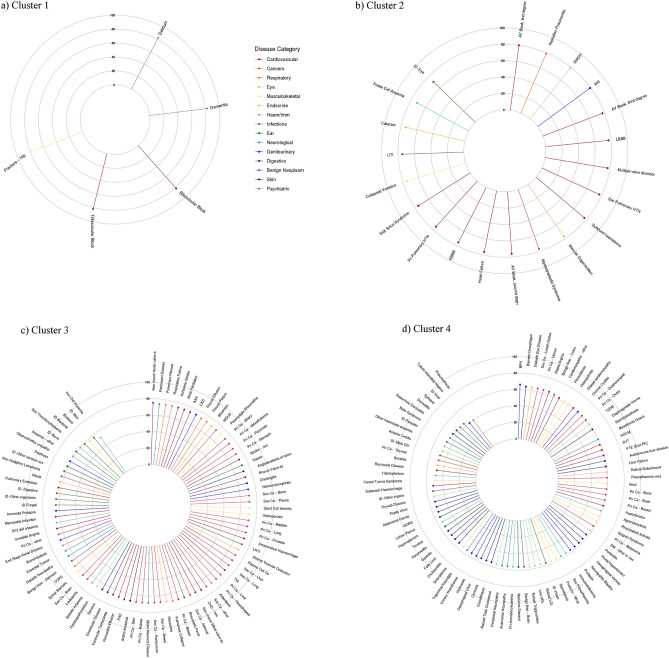
Median age of first recorded diagnosis above the age of 20 years for diseases in (**a**) Cluster 1, (**b**) Cluster 2, (**c**) Cluster 3 and (**d**) Cluster 4. Diseases are arranged in descending order of median age of first recorded diagnosis. AAA = abdominal aortic aneurysm; AKI = acute kidney injury; AV = atrioventricular; Benign Neo = benign neoplasm; CHD = coronary heart disease; CKD = chronic kidney disease; COPD = chronic obstructive pulmonary disease; DM = diabetes mellitus; dz = disease; GORD = gastroesophageal reflux disease; GU = genitourinary; HDL = high density lipoprotein cholesterol; HOCM = hypertrophic obstructive cardiomyopathy; HTN = hypertension; ID = infectious disease; LBBB = left bundle branch block; LDL = low density lipoprotein cholesterol; LRTI = lower respiratory tract infection; MGUS = monoclonal gammopathy of undetermined significance; nos = not otherwise specified; PAD = peripheral arterial disease; Pri Ca = primary cancer; RBBB = right bundle branch block; Sec Ca = secondary cancer; SIADH = syndrome of inappropriate antidiuretic hormone; SVT = supraventricular tachycardia; T2DM = type 2 diabetes; TIA = transient ischaemic attack; UTI = urinary tract infection; VTE (Excl PE) = venous thromboembolism excluding pulmonary embolism.

The median age of diagnosis above the age of 20 years for every disease in Clusters 1, 2, 3 and 4 is displayed in Fig. 5. Dementia and delirium in Cluster 1 had the highest median age of diagnosis (83y for both). Supplementary Table S4 shows the median age of diagnosis above the age of 20 years (median age, (interquartile range (IQR)) for diseases stratified by category and cluster.

### Gompertz and Gompert-Makeham models

We next employed an actuarial method to determine whether a disease was ageing-related. We developed an algorithm which applied mortality models to age-specific rates of disease onset for 289 diseases as described in Fig. 1. The Gompertz function, which is monotonic, was used to filter diseases with rates of onset that decreased with age. The goodness-of-fit of the Gompertz–Makeham (GM) model, which is exponential-polynomial, and hence may be used to fit non-monotonic curves, was assessed to evaluate whether a disease was ageing-related. Higher values of the adjusted R^2^ of the GM indicated a better fit, and therefore a higher likelihood that the disease was ageing-related.

35 conditions had a negative coefficient of the age variable in the Gompertz model (Eq. 4) and could therefore be considered to have a very low likelihood of being ageing-related. Of the remaining 254 diseases, the majority (210) had adjusted R^2^ of the GM model above 0.95, indicating a very high likelihood of being ageing-related. 193 of these 210 diseases were in Clusters 1–4 (Table 2).

**Table 2:** The number of conditions in each age-related and outlier cluster for different thresholds of adjusted R^2^ (x) (with a positive β (coefficient of the age variable) in the Gompertz model), and the number of conditions with a negative β.

Adjusted R^2^ (*x*)	β > 0 *x* > 0.95	β > 0 0.9 < *x* < 0.95	β > 0 0.85 < *x* < 0.9	β > 0 0.8 < *x* < 0.85	β > 0 *x* < 0.8	β < 0
Number of conditions	210	17	8	2	17	35
Cluster 1	5	0	0	0	0	0
Cluster 2	21	0	0	0	0	0
Cluster 3	87	4	0	0	0	0
Cluster 4	80	7	3	0	0	0
Cluster 5	16	4	4	2	13	12
Cluster 6	0	0	0	0	0	7
Cluster 7	0	0	0	0	1	2
Cluster 8	0	0	0	0	0	7
Cluster 9	0	0	0	0	0	3
Outlier 1	1	0	0	0	0	0
Outlier 2	0	1	0	0	0	0
Outlier 3	0	1	0	0	0	0
Outlier 4	0	0	0	0	1	0
Outlier 5	0	0	1	0	1	0
Outlier 6	0	0	0	0	0	1
Outlier 7	0	0	0	0	1	0
Outlier 8	0	0	0	0	0	2
Outlier 9	0	0	0	0	0	1

**Figure 6:**
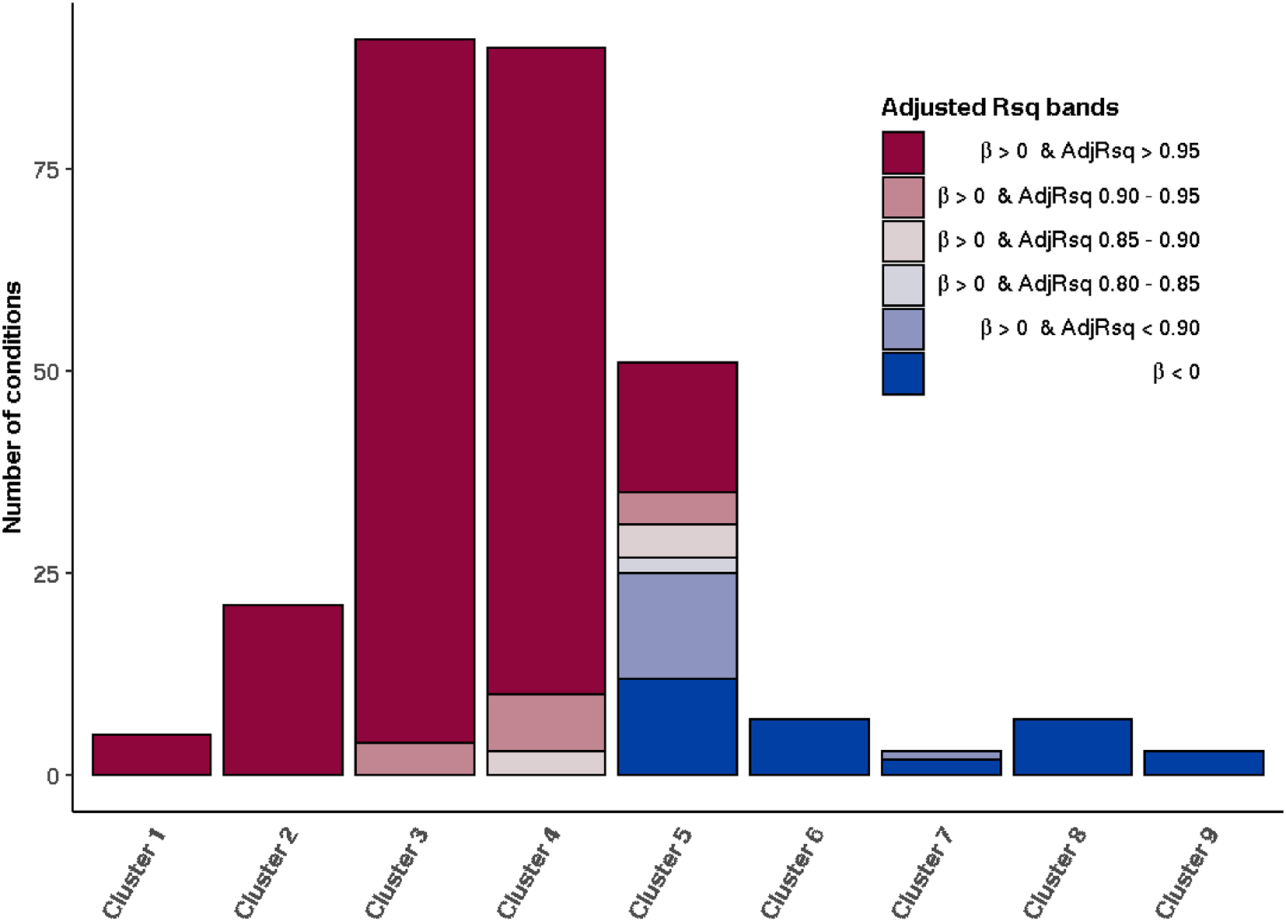
Number of diseases in each curve cluster for different adjusted R^2^ bands where *β* is positive, and number of diseases where *β* is negative. *β* is the coefficient of the age variable in the Gompertz model and the adjusted R^2^ value measures the goodness-of-fit of the Gompertz–Makeham model.

All 26 diseases in Clusters 1 and 2 had a very high likelihood of being ageing-related, with adjusted R^2^ values for the GM model above 0.95. The adjusted R^2^ of the GM model was above 0.95 for 87 out of 91 diseases in Cluster 3, and between 0.90 and 0.95 for four diseases (secondary bowel cancer, primary prostate cancer, mesothelioma and iron deficiency anaemia). Similarly, in Cluster 4, the majority of diseases had adjusted R^2^ of the GM model above 0.95 (80 out of 90 diseases), albeit a smaller proportion than in Clusters 1, 2 and 3. The adjusted R^2^ of the GM model was below 0.90 for three conditions in Cluster 4—parasitic infection (0.88021), hyposplenism (0.88019) and primary thyroid cancer (0.85776) (Table 2, Fig. 6, Supplementary Table S1).

Twelve diseases in Cluster 5 had a negative coefficient of the age variable in the Gompertz model, with a very low probability of being ageing-related. Of the remaining 39 diseases, the adjusted R^2^ of the GM model was above 0.95 for 16 conditions, between 0.90 and 0.95 for four conditions and below 0.90 for 19 conditions (Table 2, Fig. 6).

All conditions in Clusters 6 to 9 were unlikely to be ageing-related. With the exception of schizophrenia spectrum, all conditions in these clusters had a negative coefficient of the age variable for the Gompertz model. The adjusted R^2^ of the GM model for schizophrenia spectrum was 0.70529, indicating a poor fit for the GM model, had hence a very low likelihood of being ageing-related (Table 2, Fig. 6, Supplementary Table S1).

## Discussion

We grouped 278 high-burden diseases into nine main clusters using unsupervised machine-learning. Four of these clusters consisted of diseases that increased with age, albeit with strikingly different age trajectories and median ages of disease onset (82y, 77y, 69y and 57y for Clusters 1, 2, 3 and 4, respectively), indicating that different aetiologies may drive each cluster. Diseases in these four clusters spanned diverse organ systems and clinical specialties. Cluster 1 consisted of dementia, delirium, hip fracture, bifascicular and trifascicular heart blocks. Cardiovascular diseases were most highly represented in Cluster 2, cancers in Cluster 3, and diseases of the digestive system in Cluster 4. Benign neoplastic, skin and psychiatric disorders, the three disease categories with the lowest median age of disease onset (50y, 43y and 38y, respectively), were largely absent from these four clusters. Four clusters (Clusters 6, 7, 8 and 9) were clearly not ageing-related. Cluster 5 comprised diseases with varying age-related disease onset patterns.

Next, we applied actuarial techniques to assess whether diseases were ageing-related according to how well the rate of disease onset data fitted the Gompertz and Gompertz–Makeham models. While this method was based on very different principles from the clustering algorithm, the results were highly concordant (Table 2, Fig. 6) indicating that these two data-driven approaches can be used synergistically to identify ARDs.

All diseases in Clusters 1 and 2 were highly likely to be ageing-related. A small number of diseases in Clusters 3 and 4 fit slightly less well with the actuarial models. Unlike clustering techniques, parametric methods such as the Gompertz and GM models rely on sufficient sample sizes to assess how well the model fits a particular distribution. Where sample sizes are small (i.e. data is sparse), the goodness-of-fit statistics are lower, reflecting the lower degree of certainty with which the assumed model fits the data. The relationship with age for diseases in Cluster 5 was more complex than for diseases in the other clusters. Given the heterogeneity in the age-specific rate of disease onset curves in this cluster, the actuarial method was useful in differentiating diseases which were likely to be ageing-related, such as erectile dysfunction, from those that were not, such as irritable bowel syndrome (Supplementary Fig. S6).

Clustering of age density patterns of ICD-10 codes on medical claims from an insurance company in Brazil has been described previously^28^, but to our knowledge, this is the first report of clustering of age-specific rates of disease onset of curated disease phenotypes in a representative population set, with the results corroborated using an independent parametric method, namely actuarial models. Unlike data from a universal healthcare system such as the National Health Service (NHS) in England, insurance claims data may be biased and not representative of a population of interest as they exclude individuals without health insurance, and data collected primarily for financial purposes may not be suitable to assess epidemiological measures such as prevalence and incidence of disease^29,30^. Furthermore, the previous study did not provide details of which ICD-10 codes fell into each cluster, while in this study we present the age-specific rate of onset curves for 289 diseases and their respective clusters so that readers can observe how disease incidence progresses with age.

In its latest version of the International Classification of Diseases, ICD-11, the World Health Organisation (WHO) has implemented an extension code for “ageing-related” diseases (XT9T), defined as those “caused by pathological processes which persistently lead to the loss of organism’s adaptation and progress in older ages”^31^. This study provides an objective method for identifying candidate diseases to which this extension can be applied.

The ARDs we identified extend across the full range of conventional classifications of disease, which are based on organ systems, as reflected in the International Classification of Diseases. We introduce an alternative paradigm for the classification of ARDs based on the age of disease onset patterns. The analytic approaches in this study can be applied to any of the thousands of phenotyped health conditions in any representative population setting to identify and categorise ARDs according to the relationship between age and rate of disease onset. Our findings facilitate the organisation of clinical specialties, particularly geriatric medicine, around the prevention or care of clusters of ARDs.

The identification of ARDs, and the presentation of age incidence curves in particular, enable clinicians to assess the likelihood of different diseases occurring at different ages. This information can be used to formulate a list of differential diagnoses when assessing individual patients. Conditions in Cluster 1 such as dementia, delirium and hip fracture were more likely to occur in the most elderly patients, while conditions in Cluster 2, consisting mainly of cardiovascular diseases, occurred at a slightly younger age, and those in Cluster 3, such as cancers, occurred earlier yet. These findings have resource implications as well. Health care providers will need to allocate more resources to diseases in Clusters 1 and 2 as populations get older. These include increased funding towards social care and allied health professional support such as physiotherapists and occupational therapists to address the functional implications of cognitive loss in dementia. These findings should also prompt increased provision of cardiac rehabilitation services to improve the quality of life of individuals who experience heart failure and arrhythmias as a result of insults to the cardiovascular system at an earlier age. Our results can also guide health services to target preventive measures for ARDs in the different clusters at different ages over the lifecourse, such as providing occupational health assessments for individuals above the age of 80 years to prevent falls leading to hip fractures. The findings from this study also give basic science researchers a perspective on the incidence of ARDs over the lifecourse and demonstrate which ARDs have similar patterns of disease onset with age, thereby informing research into how long various hallmarks or mechanisms of ageing may take to cause ARDs in the different clusters. Future research is needed to investigate whether diseases in the same cluster share common mechanisms or risk factors of ageing.

ARDs that occur together more often than expected by chance may share common biological mechanisms. If so, existing drugs targeting these mechanisms could be repurposed for other ARDs with similar molecular pathways. For example, interleukin 6 (IL6), an inflammatory cytokine, has been implicated in the pathogenesis of rheumatoid arthritis^32^, coronary heart disease^33^, atrial fibrillation^34^ and abdominal aortic aneurysm^35^. Drugs such as tocilizumab, which inhibits the IL6-receptor and is already licensed for the treatment of rheumatoid arthritis and giant cell arteritis, might therefore be effective in treating these other diseases. New drugs can also be developed to modulate the biological pathways for multiple ARDs based on common genetic or other molecular risk factors.

ARDs such as alcoholic liver disease, COPD, cirrhosis, cancers, peptic ulcer, and actinic keratosis are caused by the cumulative damage of exogenous substances including alcohol, smoking, medications, deleterious dietary compounds, and radiation. Research into environmental causes and public health campaigns that target these are important to prevent ARDs amenable to lifestyle and public policy changes.

We identified ARDs using methods that relied on large population EHR datasets. Replication in independent representative population cohorts would validate the application of these methods to big data with defined disease phenotypes (not just ICD-10 or other billing codes) from other healthcare systems that are representative of the general population. This would pave the way to comparisons of how diseases may vary with age across high, medium and low-income countries, and countries with different population age structures.

One potential limitation of our analysis was that the age of disease onset was represented by the age of first recorded diagnosis for each individual^11^. This could introduce biases in the rate of disease onset for several reasons. Diseases such as chronic obstructive pulmonary disease (COPD) are clinically silent for long periods, leading to delays between each of the following events: disease onset, presentation to a clinician, diagnosis and documentation in the EHR. Other conditions such as hypertension, dyslipidaemia or obesity were more likely to be diagnosed in individuals aged 40–74 years because of the NHS Health Checks programme which began in 2009 with the aim of reducing CVD risks^36^. Conditions that are usually asymptomatic, such as chronic kidney disease, were more likely to be detected in individuals already diagnosed with co-existing morbidities than in individuals having no contact with health services. Other factors, such as screening, may also affect recorded diagnosis rates. An example is breast cancer, where small spikes in the rate of disease onset curve are apparent at the ages of 50 and 70, which correspond to the ages between which breast screening takes place (Supplementary Fig. S5a). However, given that disease onset is often latent with minimal clinical features, and that diagnosis from clinical manifestation in this current age of medicine in high-income countries such as England is usually time-efficient, EHRs present us with the best available proxy for age of disease onset, for the widest spectrum of disease, in the form of age at first recorded diagnosis.

Variable patterns of consultation could also affect the accuracy of the records. Disease frequency estimates for conditions which can be self-managed by over-the-counter medications or conditions affecting individuals at the mild end of the symptom spectrum may be underestimated using EHRs. Another limitation of this study is that we did not use free text comments to supplement the phenotyping algorithms for disease definition. This could have led to missing diagnoses for conditions that might not be well coded^37^. However, studies have shown that most diseases, including cancers, inflammatory bowel diseases, asthma, cataract, glaucoma and autism are reliably captured using diagnosis codes in primary care CPRD data linked to HES secondary care data^38–43^. Finally, we did not evaluate the data quality of the CPRD linked dataset^44^, but the use of diagnostic codes in the CPRD dataset for research purposes has previously been validated^14,45^.

In conclusion, we have developed a protocol to identify and classify ARDs from any EHR dataset representative of the general population. Our findings can be used to explore which ARDs co-occur more often than expected by chance and the common endogenous or environmental drivers behind them, leading to further research investigating the most suitable interventions to prevent or treat multiple ARDs effectively. This work is therefore the first, critical step towards tackling the challenges of ageing and ARDs, which are emerging as costly afflictions in the modern world.

## Supplementary Information

Supplementary Information.

## Data Availability

The data that support the findings of this study are available from CPRD and access is subject to approval from an Independent Scientific Advisory Committee (ISAC). The data were used under license for the current study, and so are not publicly available.

## Code availability

The code is available upon request from the corresponding author.
