# Supplementary material for: Data-driven identification of ageing-related diseases from electronic health records
Source: Sci Rep. 2021 Feb 3;11:2938. doi: 10.1038/s41598-021-82459-y (PMC7859412; doi:10.1038/s41598-021-82459-y)
Supplement: Supplementary file 1 — Supplementary Information. [file 41598_2021_82459_MOESM1_ESM.docx]

**Data-driven identification of ageing-related diseases from Electronic Health Records**

Valerie Kuan^1,2,3*^, Helen C Fraser^4^, Melanie Hingorani^5^, Spiros Denaxas^1,2,3,6^, Arturo Gonzalez-Izquierdo^1,2^, Kenan Direk^1,2^, Dorothea Nitsch^7^, Rohini Mathur^7^, Constantinos A Parisinos^1^, R Thomas Lumbers^1,2,3,8^, Reecha Sofat^1,2,3^, Ian CK Wong^9,10^, Juan P Casas^11,12^, Janet M Thornton^13^, Harry Hemingway^1,2,3,14^, Linda Partridge^#4,15^, Aroon D Hingorani^#2,3,16^

^1^ Institute of Health Informatics, University College London, UK

^2^ Health Data Research UK London, University College London, UK

^3^ University College London British Heart Foundation Research Accelerator, London, UK

^4^ Institute of Healthy Ageing, Department of Genetics, Evolution and Environment, University College London, London, UK

^5^ Moorfields Eye Hospital, London. UK

^6^ Alan Turing Institute, London, UK

^7^ Department of Non-communicable Disease Epidemiology, London School of Hygiene and Tropical Medicine, London, UK

^8^ Barts Heart Centre, St Bartholomew's Hospital, London, UK

^9^ School of Pharmacy, University College London, London, WC1N 1AX, UK

^10^ Centre for Safe Medication Practice and Research, Department of Pharmacology and Pharmacy, The University of Hong Kong, Pok Fu Lam, Hong Kong

^11^ Department of Medicine, Brigham and Women’s Hospital, Harvard Medical School, Boston, MA

^12^ Massachusetts Veterans Epidemiology Research and Information Center (MAVERIC), VA Boston Healthcare System, Boston, MA, USA

^13^ European Bioinformatics Institute, Wellcome Trust Genome Campus, Hinxton, Cambridge CB10 1SD, United Kingdom

^14^The National Institute for Health Research University College London Hospitals Biomedical Research Centre, University College London, London, W1T 7DN, UK

^15^ Max Planck Institute for Biology of Ageing, Cologne, Germany

^16^ Institute of Cardiovascular Science, University College London, UK

*Correspondence to: Valerie Kuan ([v.kuan@ucl.ac.uk](mailto:v.kuan@ucl.ac.uk))

#These authors contributed equally and jointly supervised this work

Supplementary Figure S1. Age-specific rate at first recorded diagnosis for diseases in Outlier Clusters.


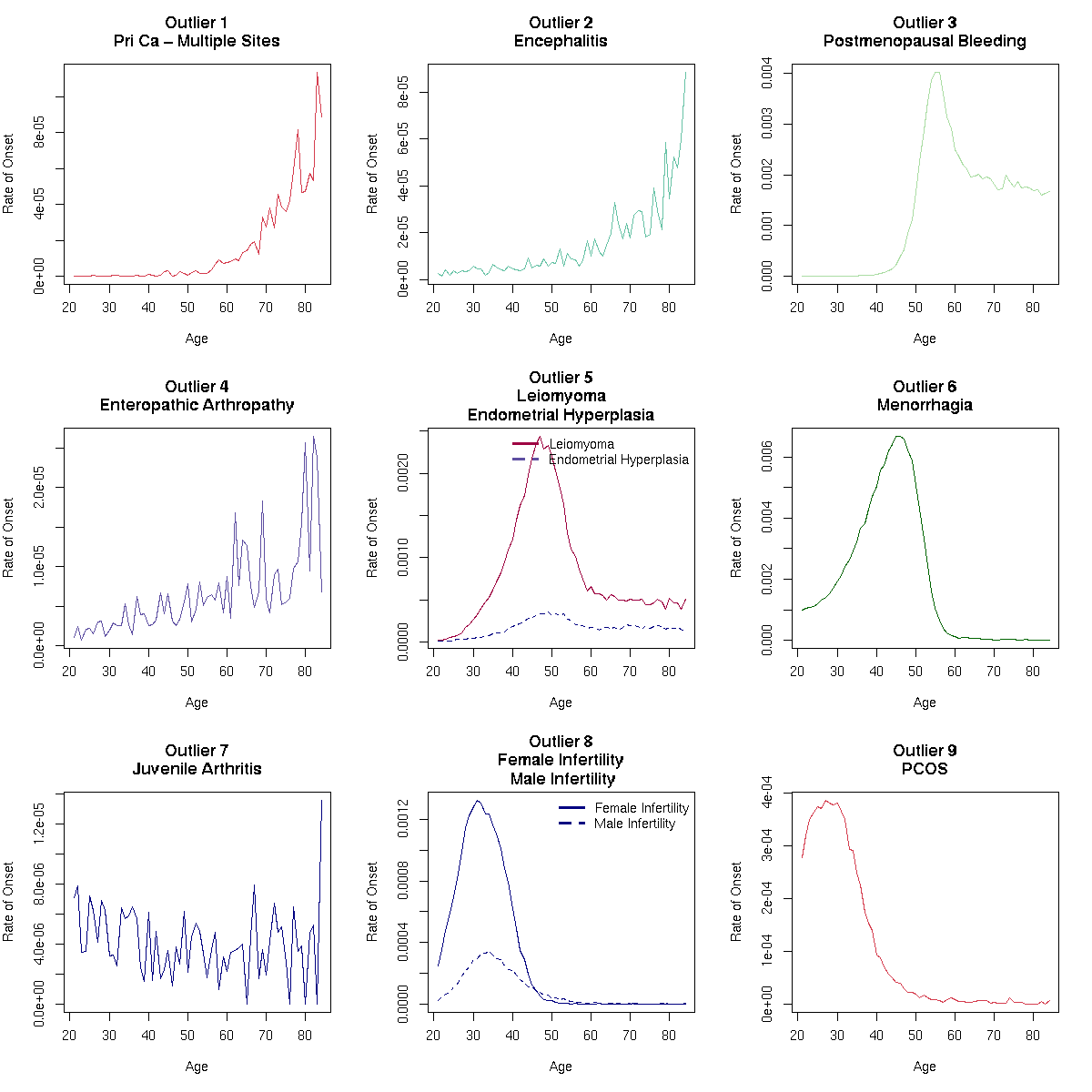


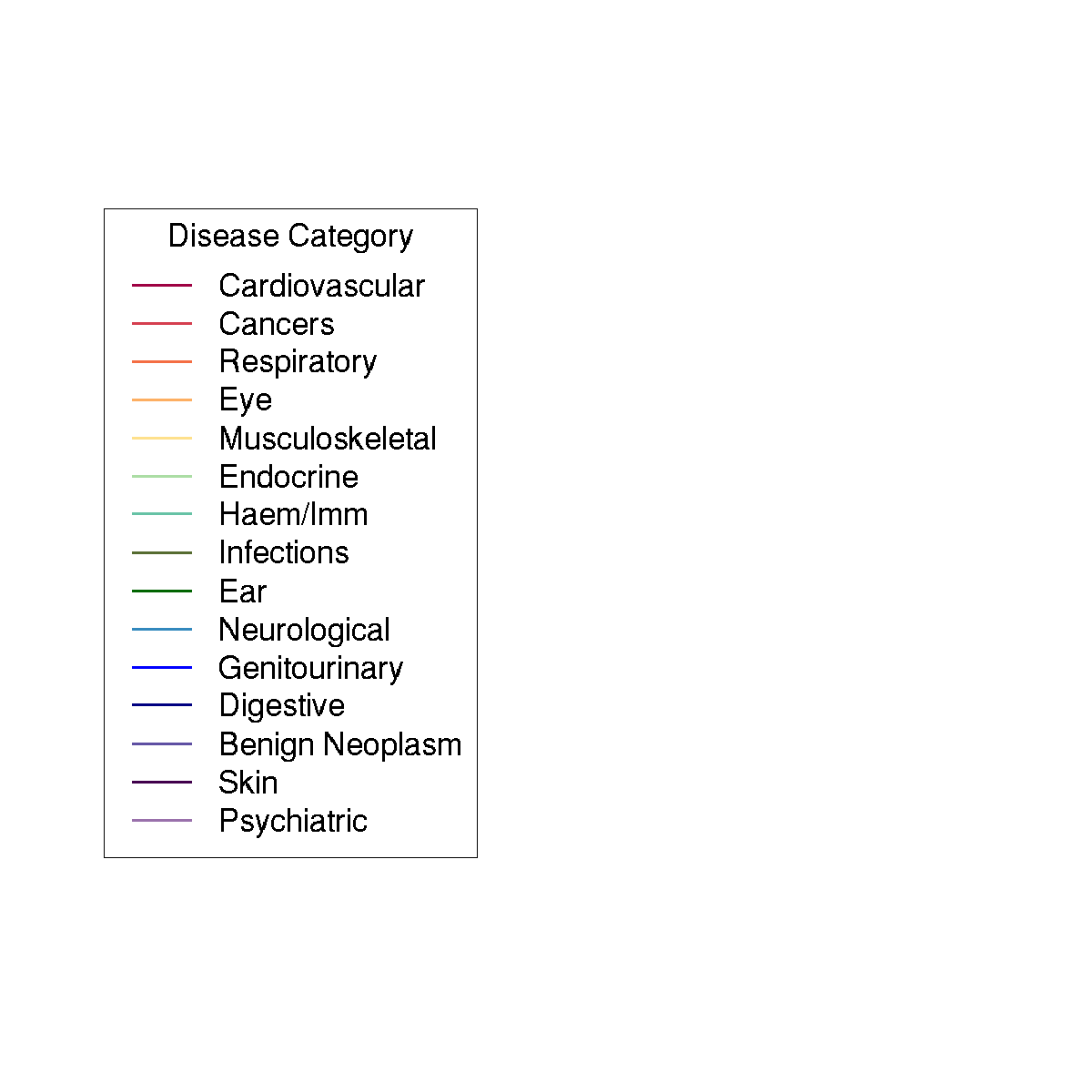


Supplementary Figure S2. Age-specific rate at first recorded diagnosis for diseases in Cluster 1.


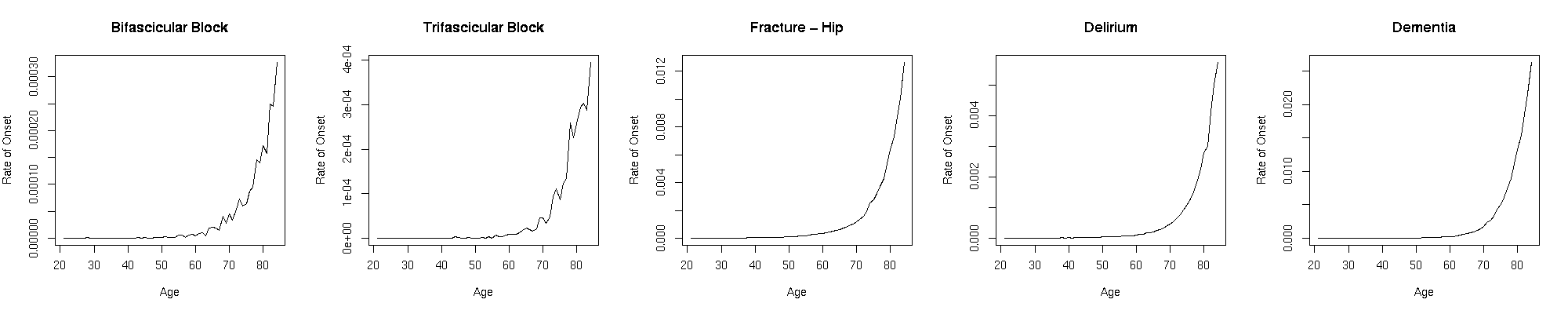


Supplementary Figure S3. Age-specific rate at first recorded diagnosis for diseases in Cluster 2.


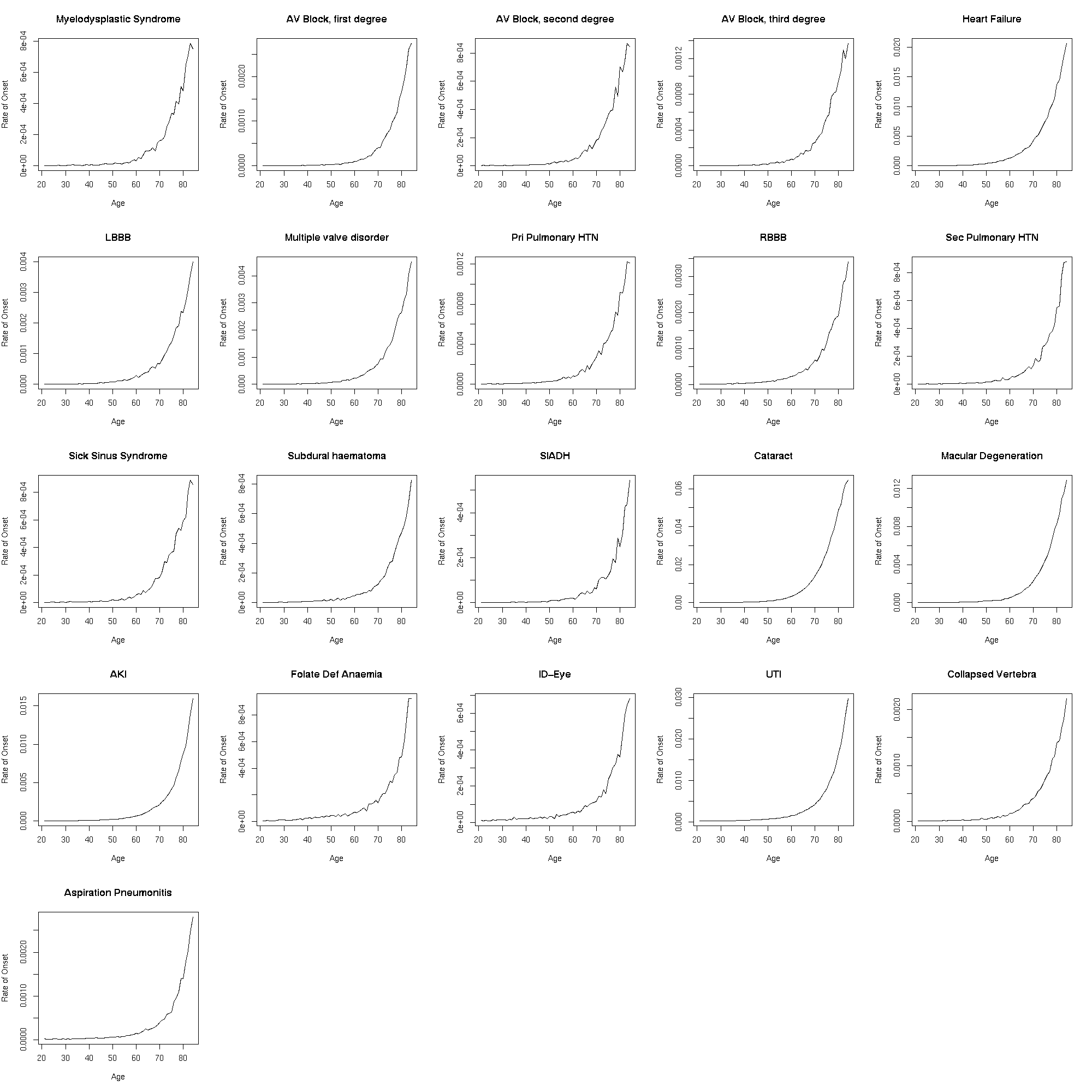


Supplementary Figure S4a. Age-specific rate at first recorded diagnosis for diseases in Cluster 3 with adjusted R^2^ of the GM model > 0.95


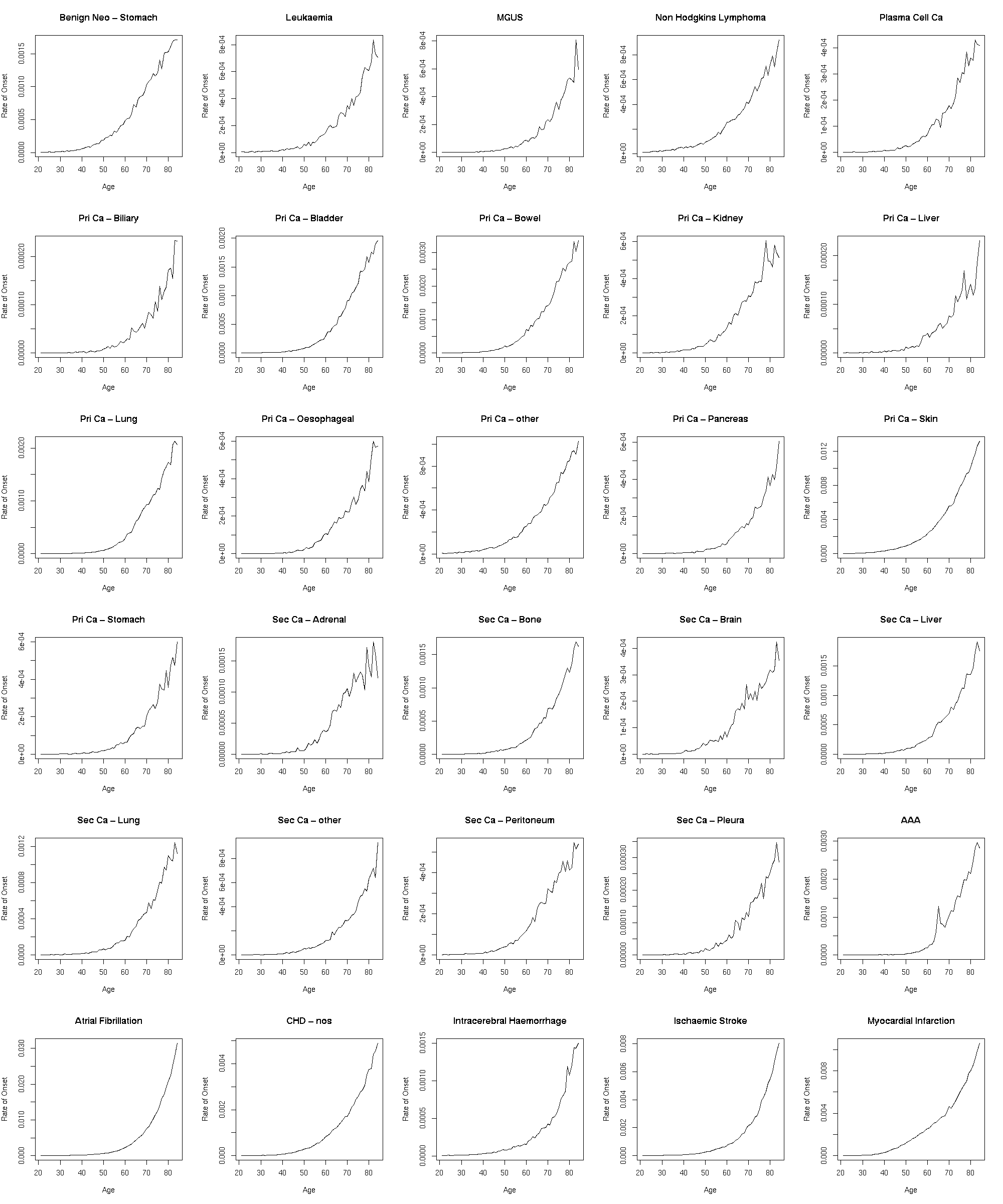


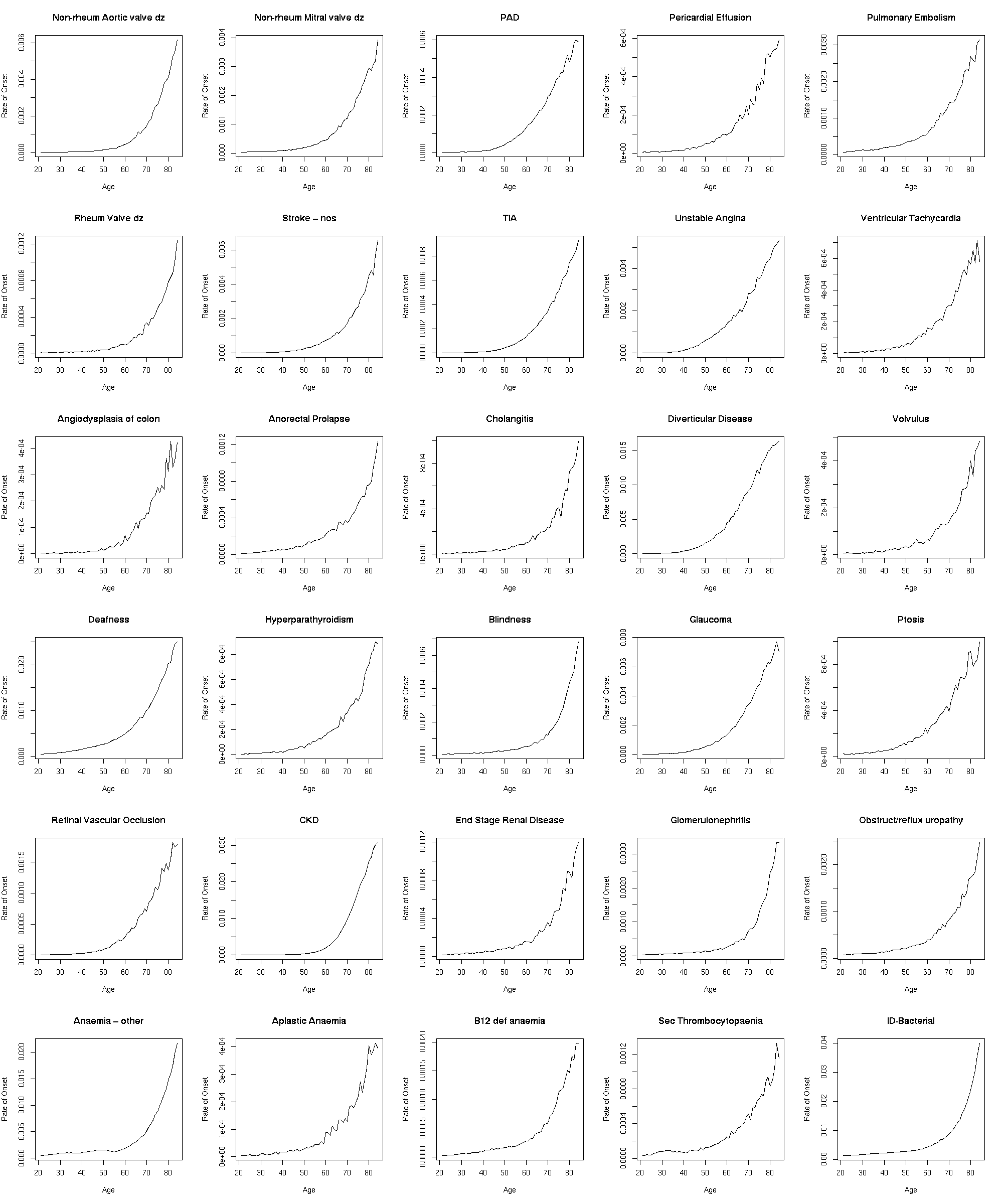


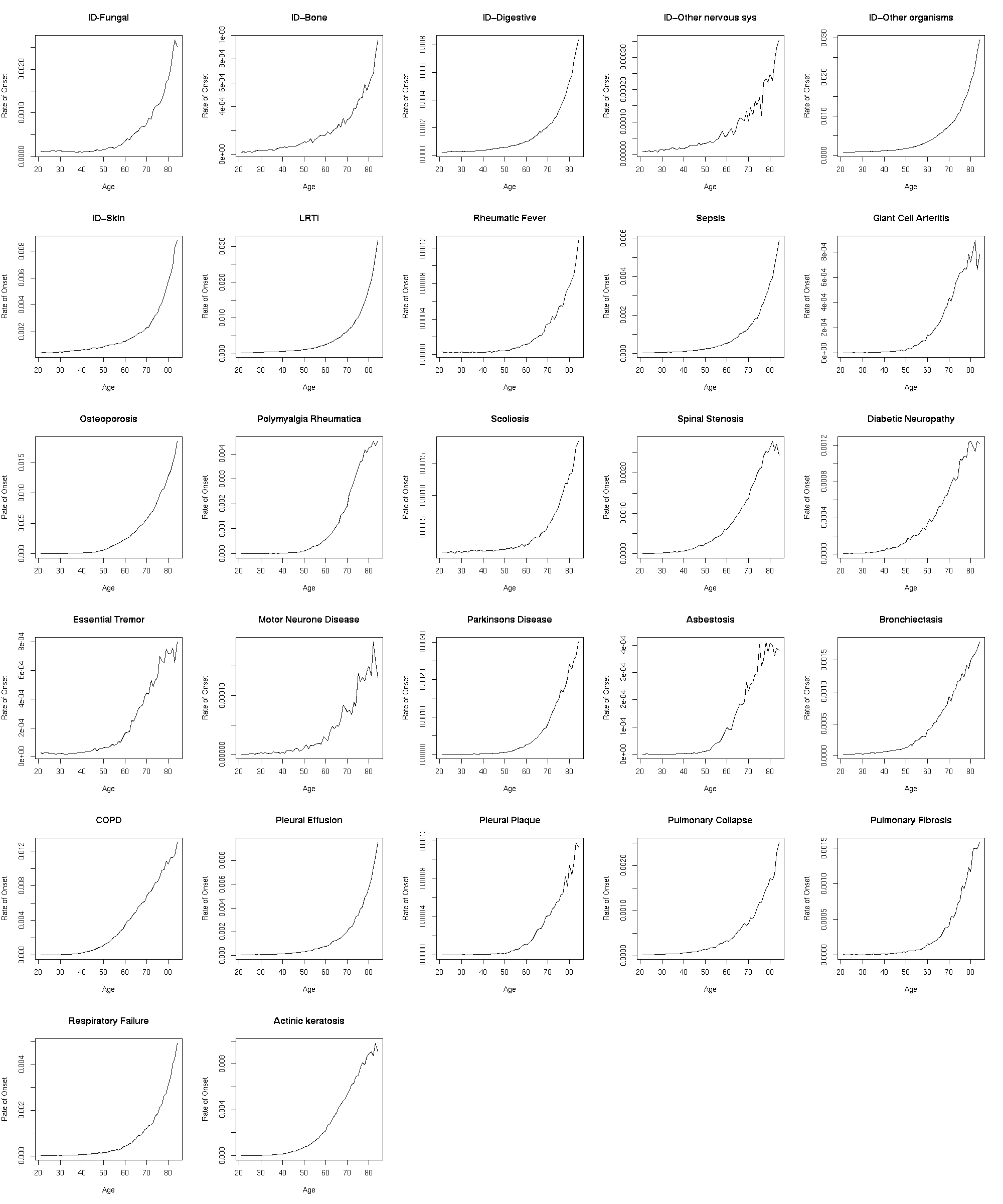


Supplementary Figure S4b. Age-specific rate at first recorded diagnosis for diseases in Cluster 3 with adjusted R^2^ of the GM model between 0.90 and 0.95


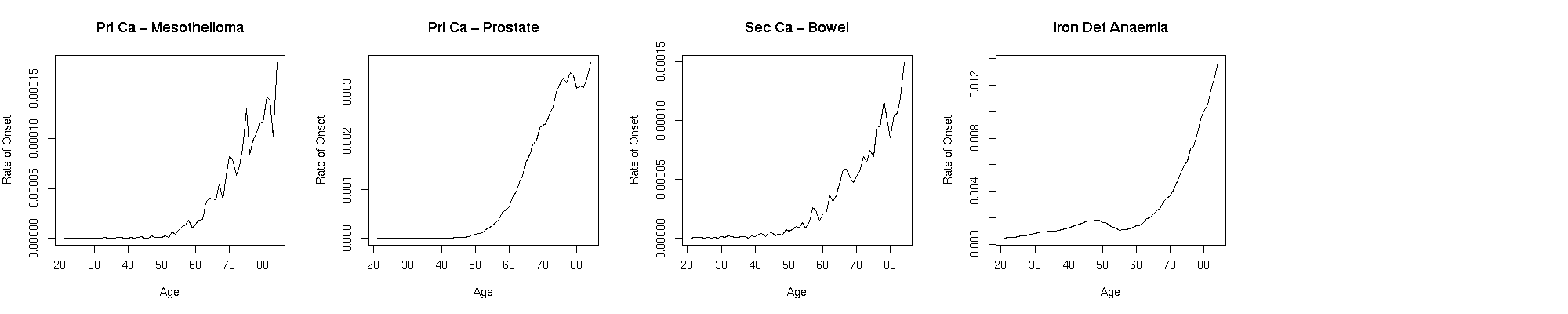


Supplementary Figure S5a. Age-specific rate at first recorded diagnosis for diseases in Cluster 4 with adjusted R^2^ of the GM model > 0.95


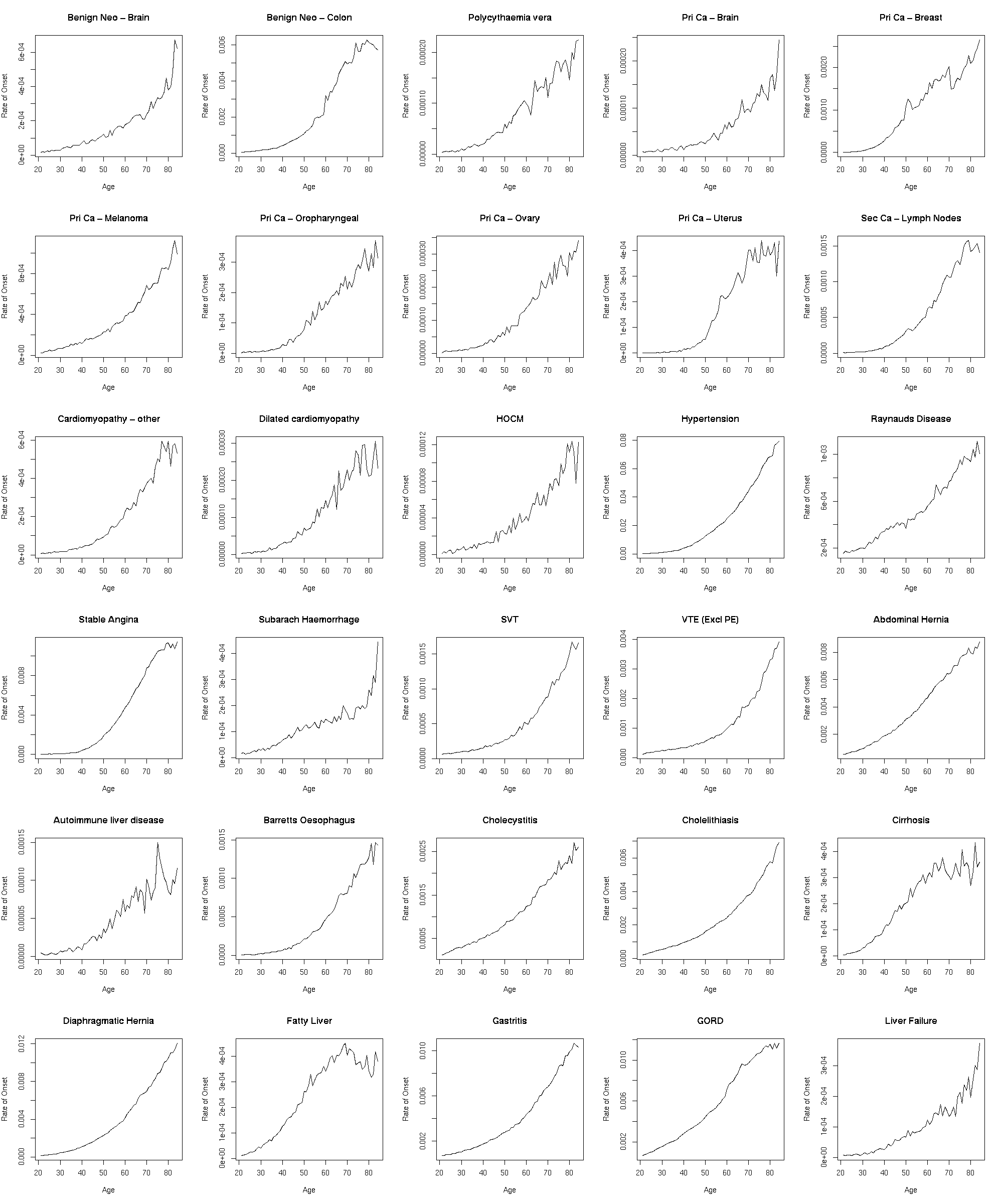


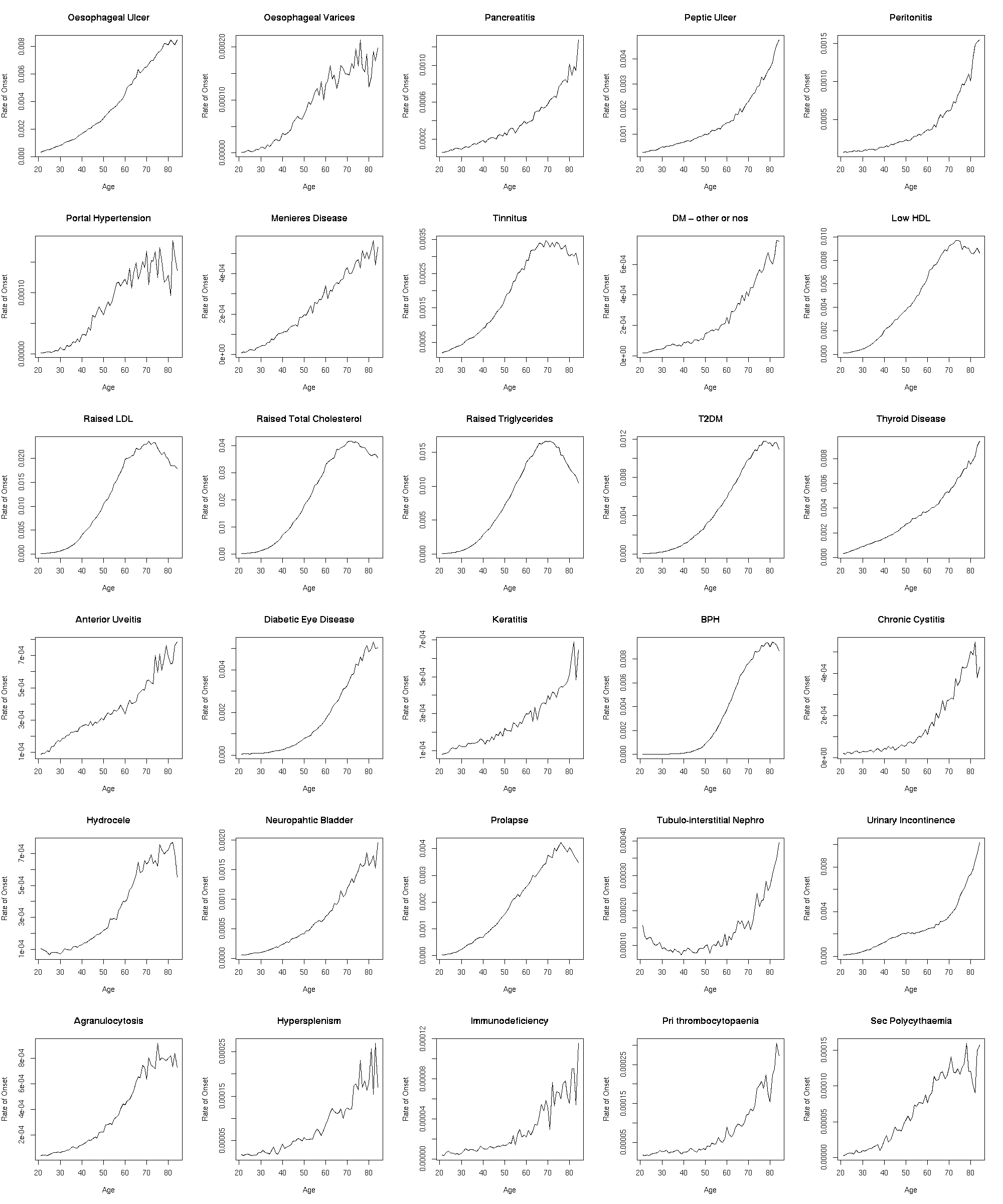


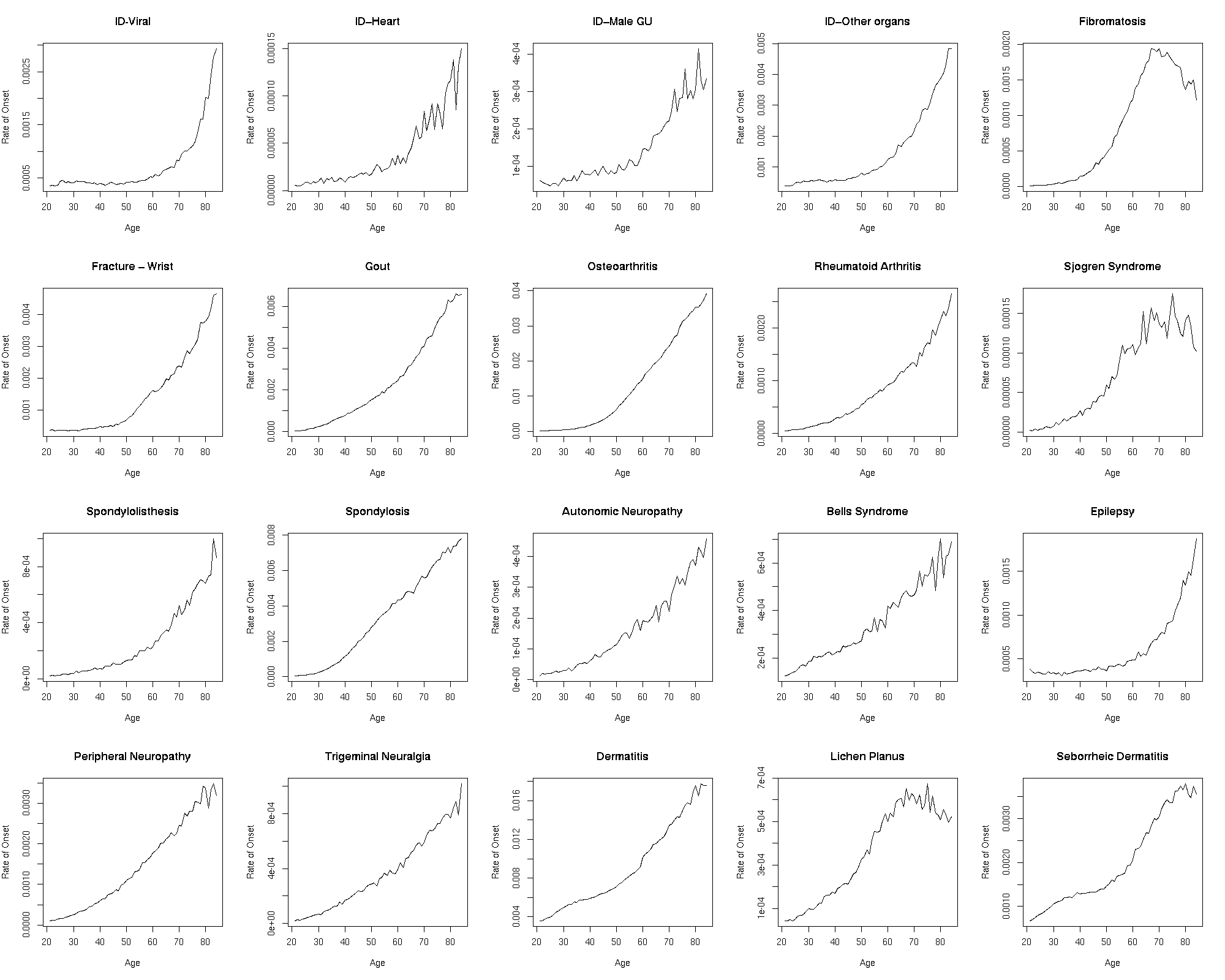


Supplementary Figure S5b. Age-specific rate at first recorded diagnosis for diseases in Cluster 4 with adjusted R^2^ of the GM model between 0.90 and 0.95


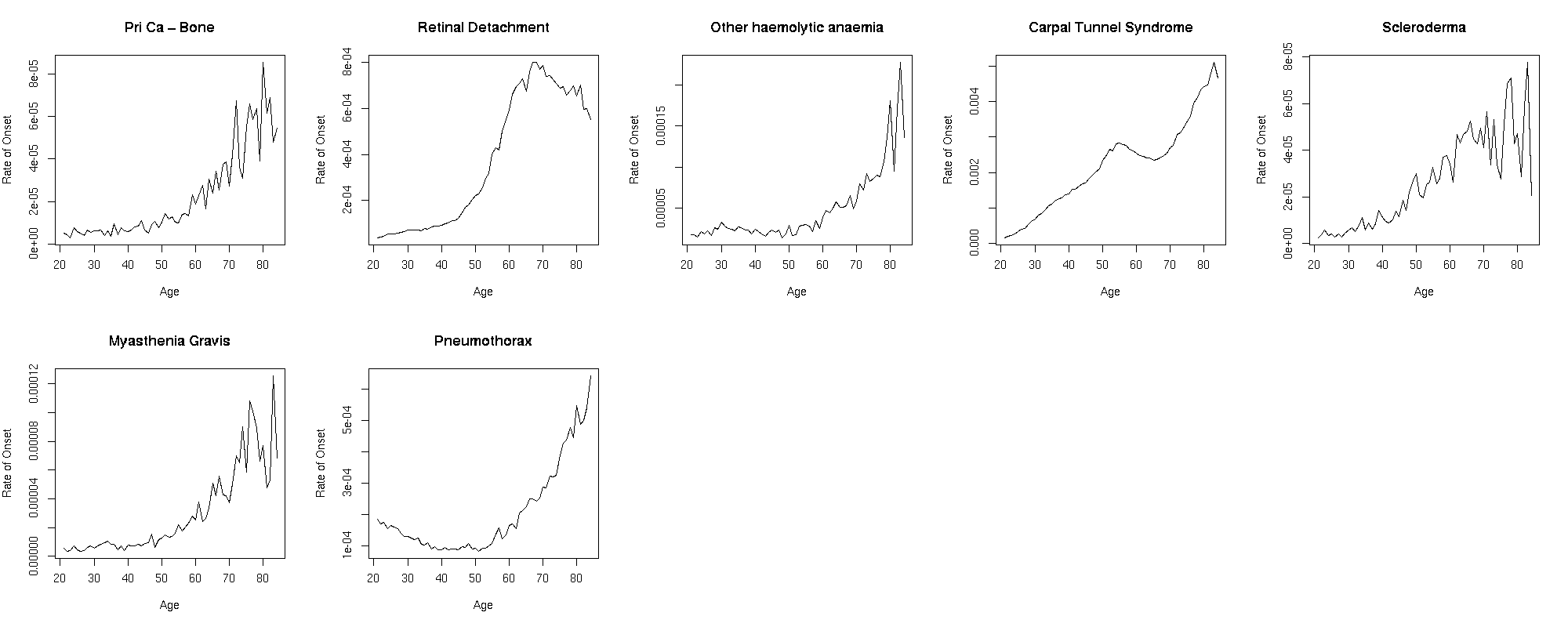


Supplementary Figure S5c. Age-specific rate at first recorded diagnosis for diseases in Cluster 4 with adjusted R^2^ of the GM model < 0.90.


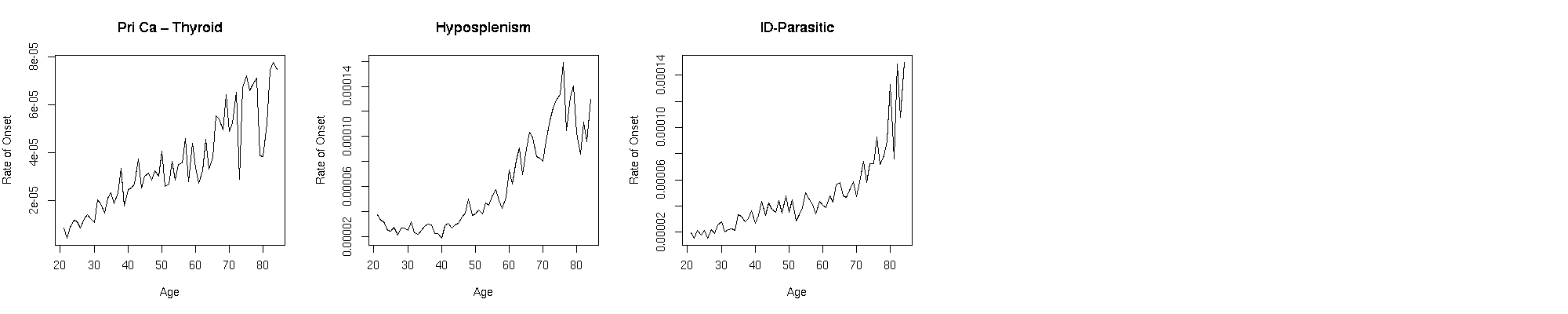


Supplementary Figure S6a. Age-specific rate at first recorded diagnosis for diseases in Cluster 5 with adjusted R^2^ of the GM model > 0.95


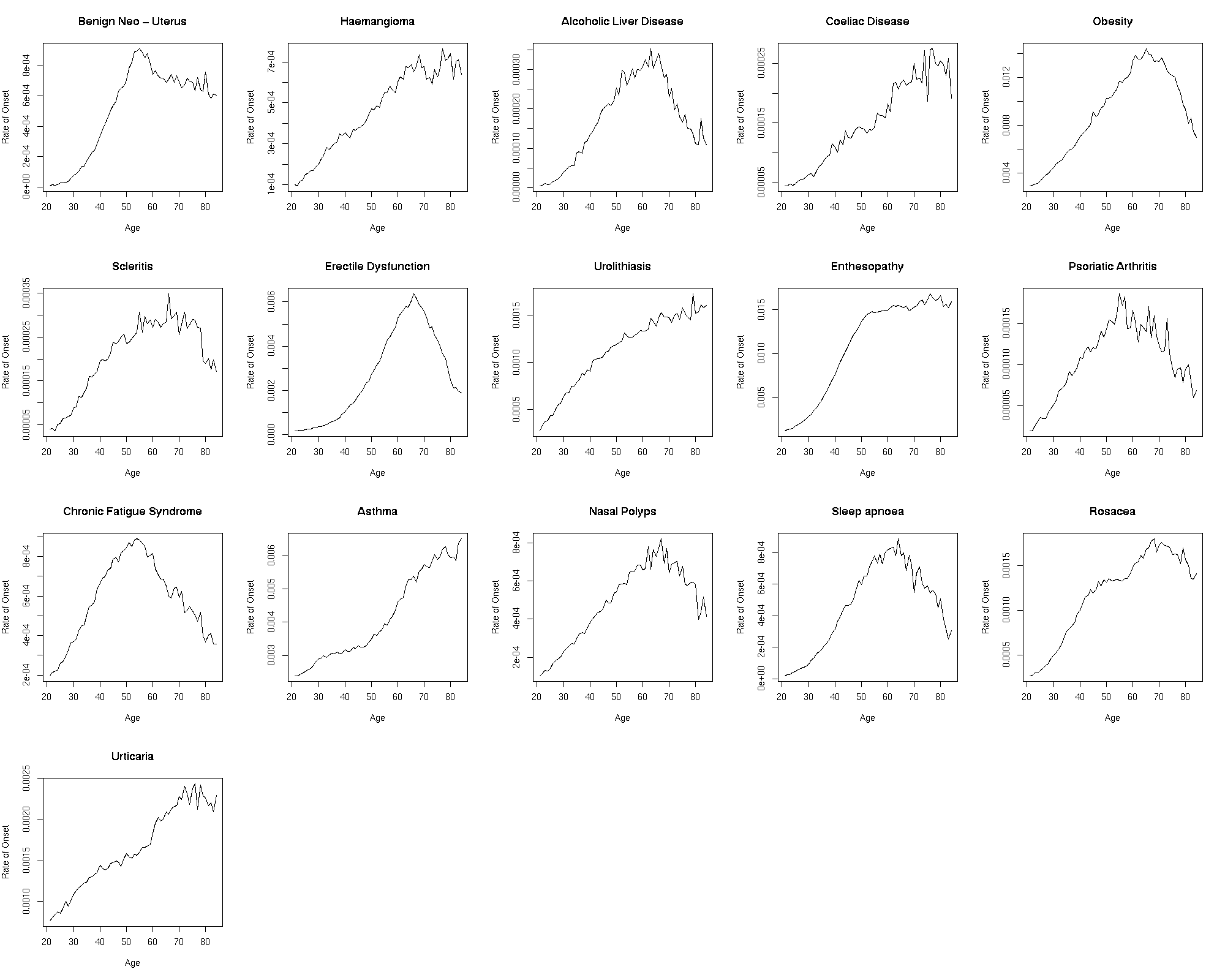


Supplementary Figure S6b. Age-specific rate at first recorded diagnosis for diseases in Cluster 5 with adjusted R^2^ of the GM model between 0.90 and 0.95


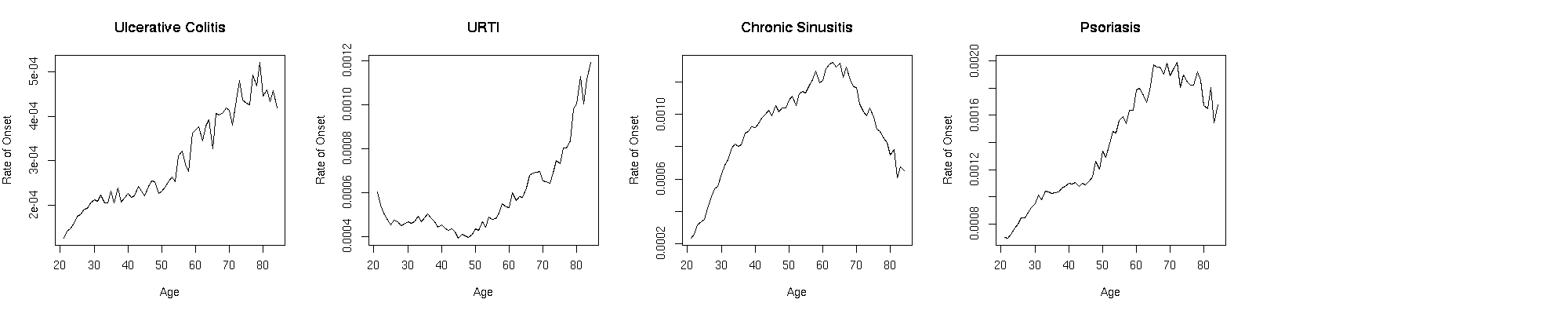


Supplementary Figure S6c. Age-specific rate at first recorded diagnosis for diseases in Cluster 5 with adjusted R^2^ of the GM model < 0.90.


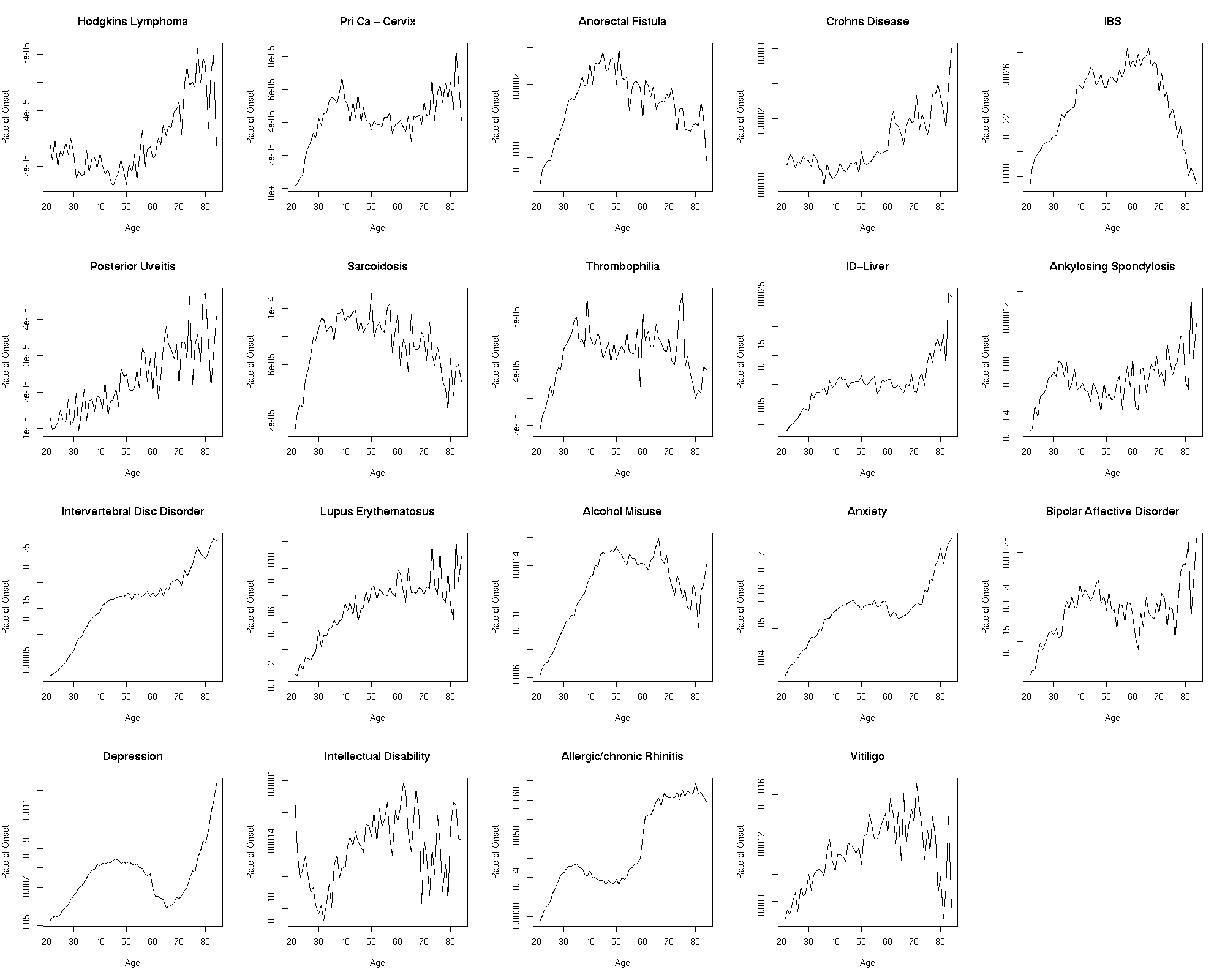


Supplementary Figure S6d. Age-specific rate at first recorded diagnosis for diseases in Cluster 5 with β (coefficient of the age term in the Gompertz model) < 0


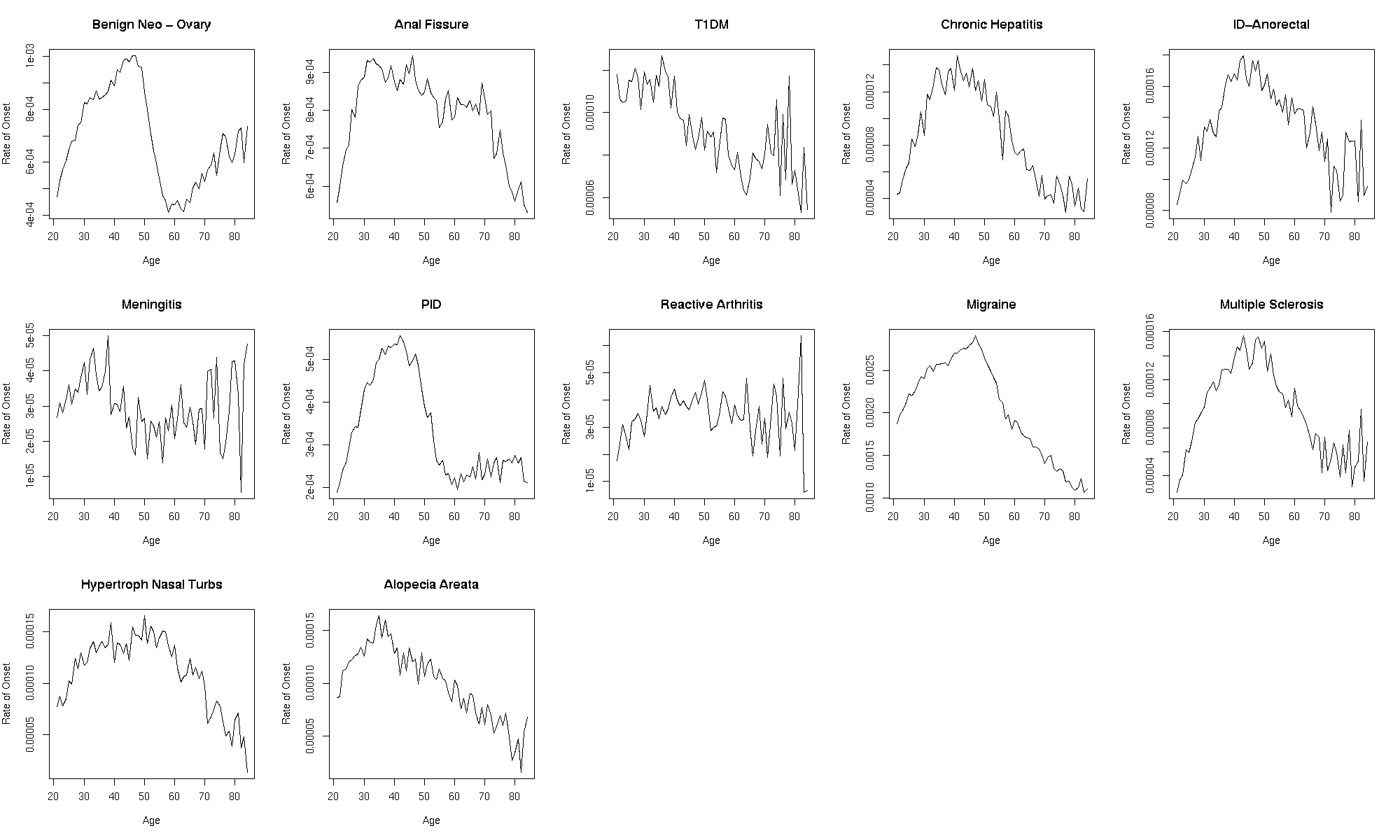


Supplementary Figure S7. Age-specific rate at first recorded diagnosis for diseases in Cluster 6.


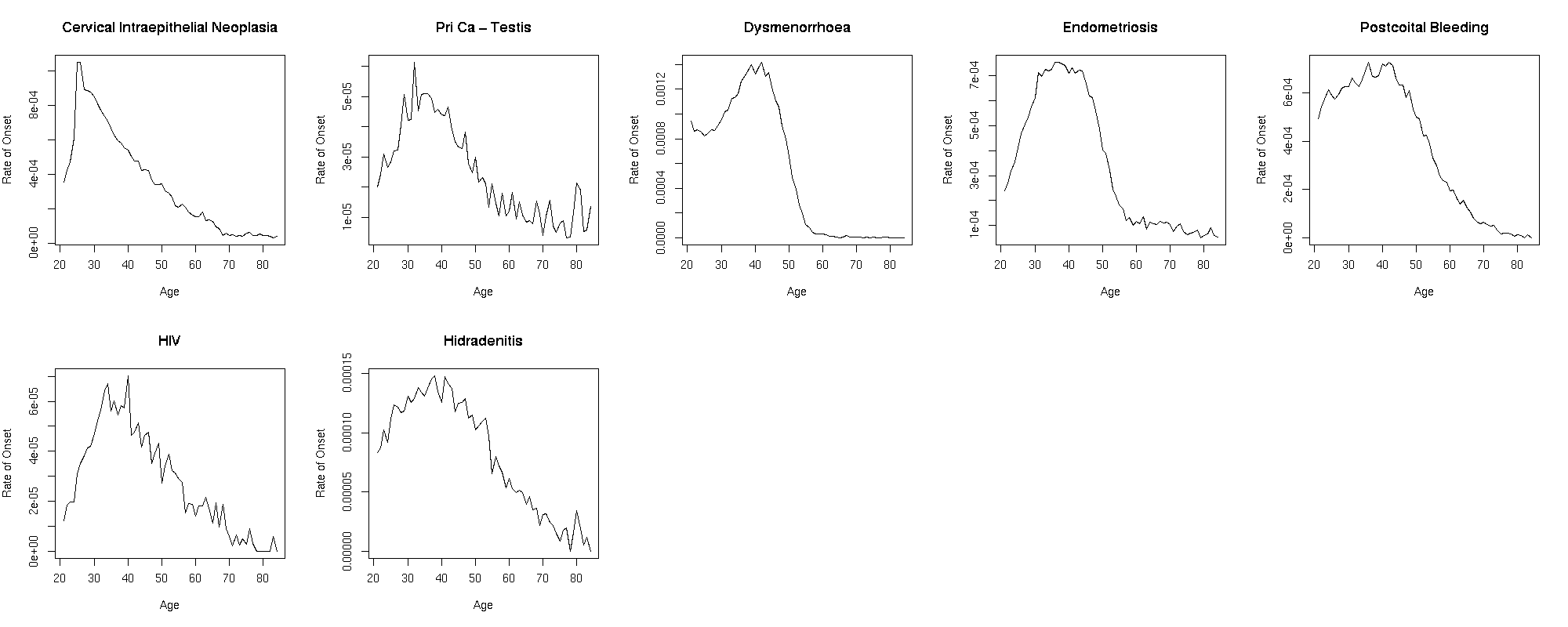


Supplementary Figure S8. Age-specific rate at first recorded diagnosis for diseases in Cluster 7.


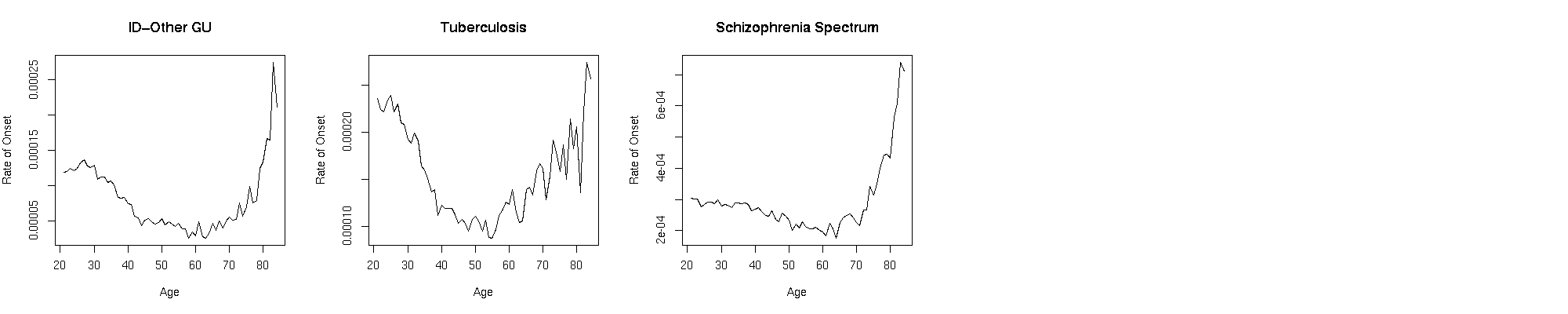


Supplementary Figure S9. Age-specific rate at first recorded diagnosis for diseases in Cluster 8.


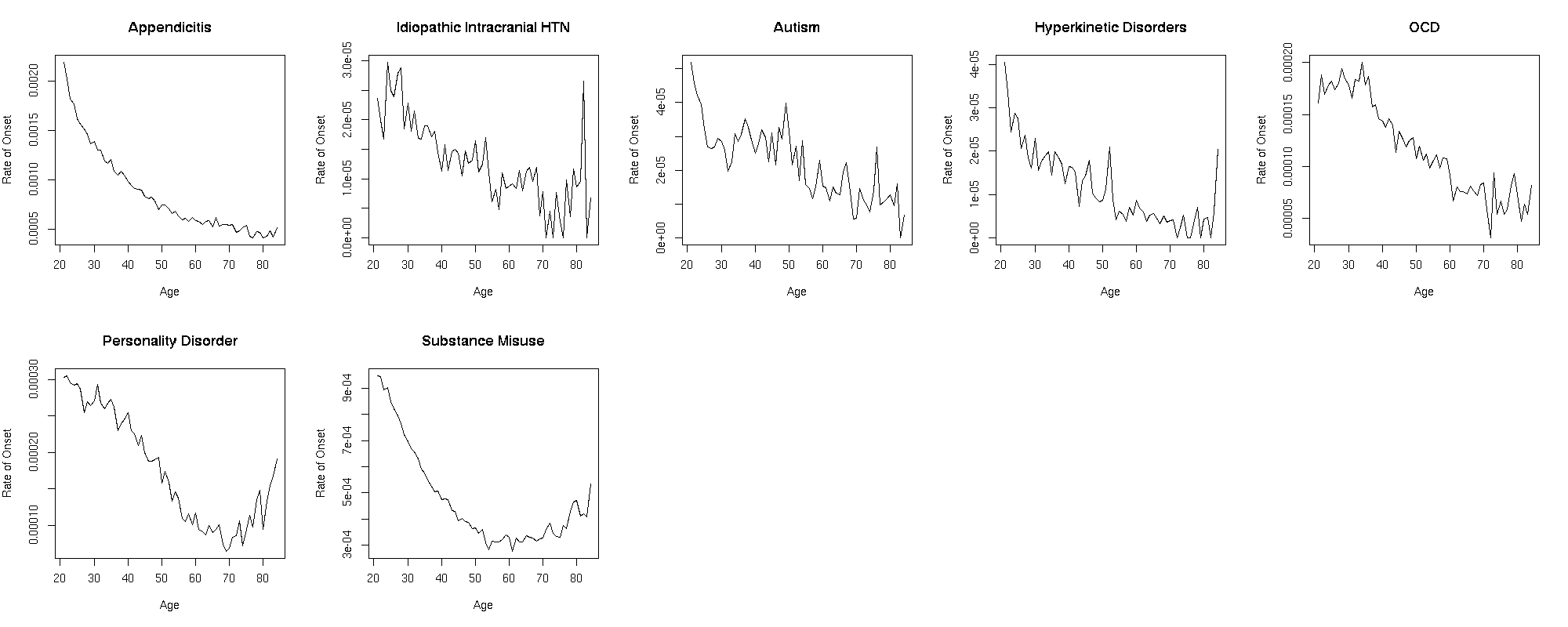


Supplementary Figure S10. Age-specific rate at first recorded diagnosis for diseases in Cluster 9.


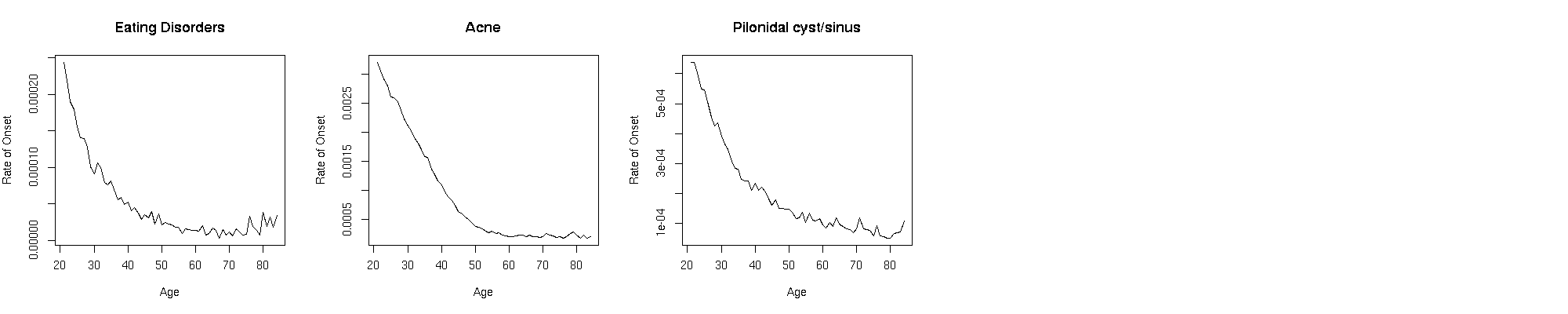


Supplementary Figure S11. Subdendrogram showing the result of the hierarchical agglomerative clustering algorithm for Cluster 1.


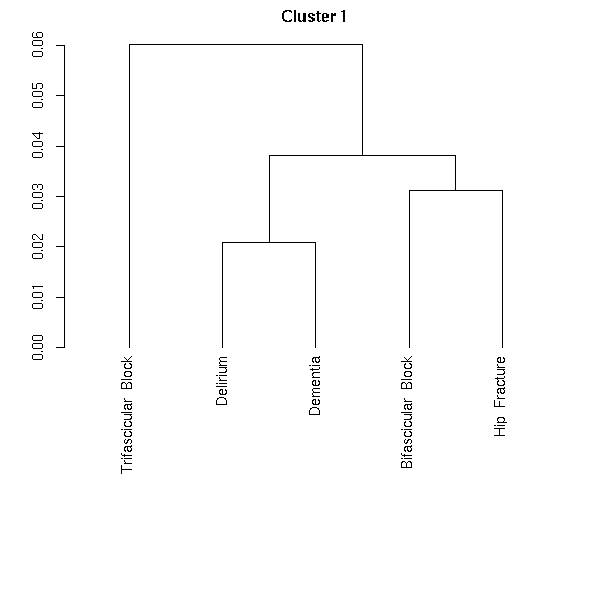


Supplementary Figure S12. Subdendrogram showing the result of the hierarchical agglomerative clustering algorithm for Cluster 2.


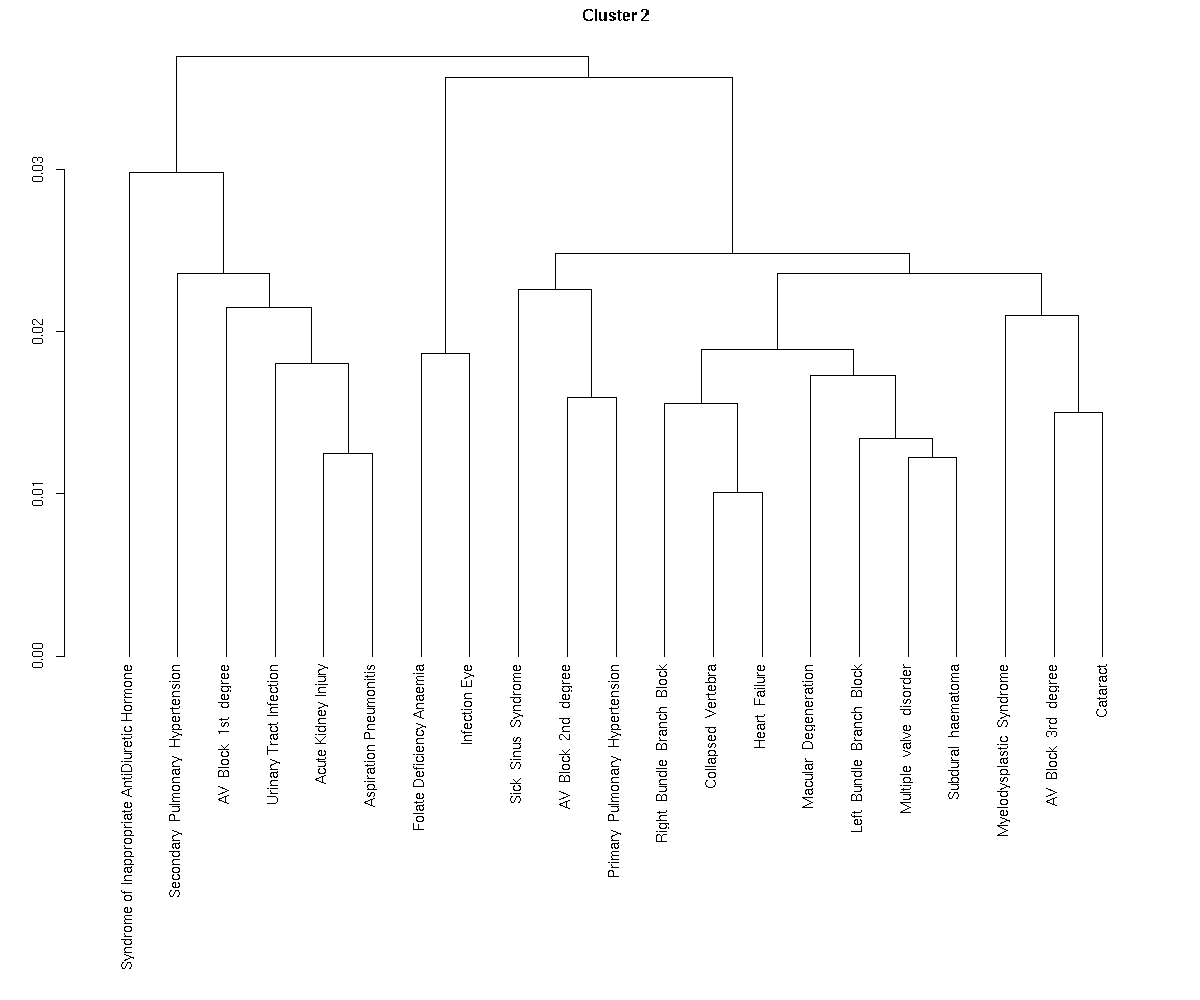


Supplementary Figure S13. Subdendrogram showing the result of the hierarchical agglomerative clustering algorithm for Cluster 3.


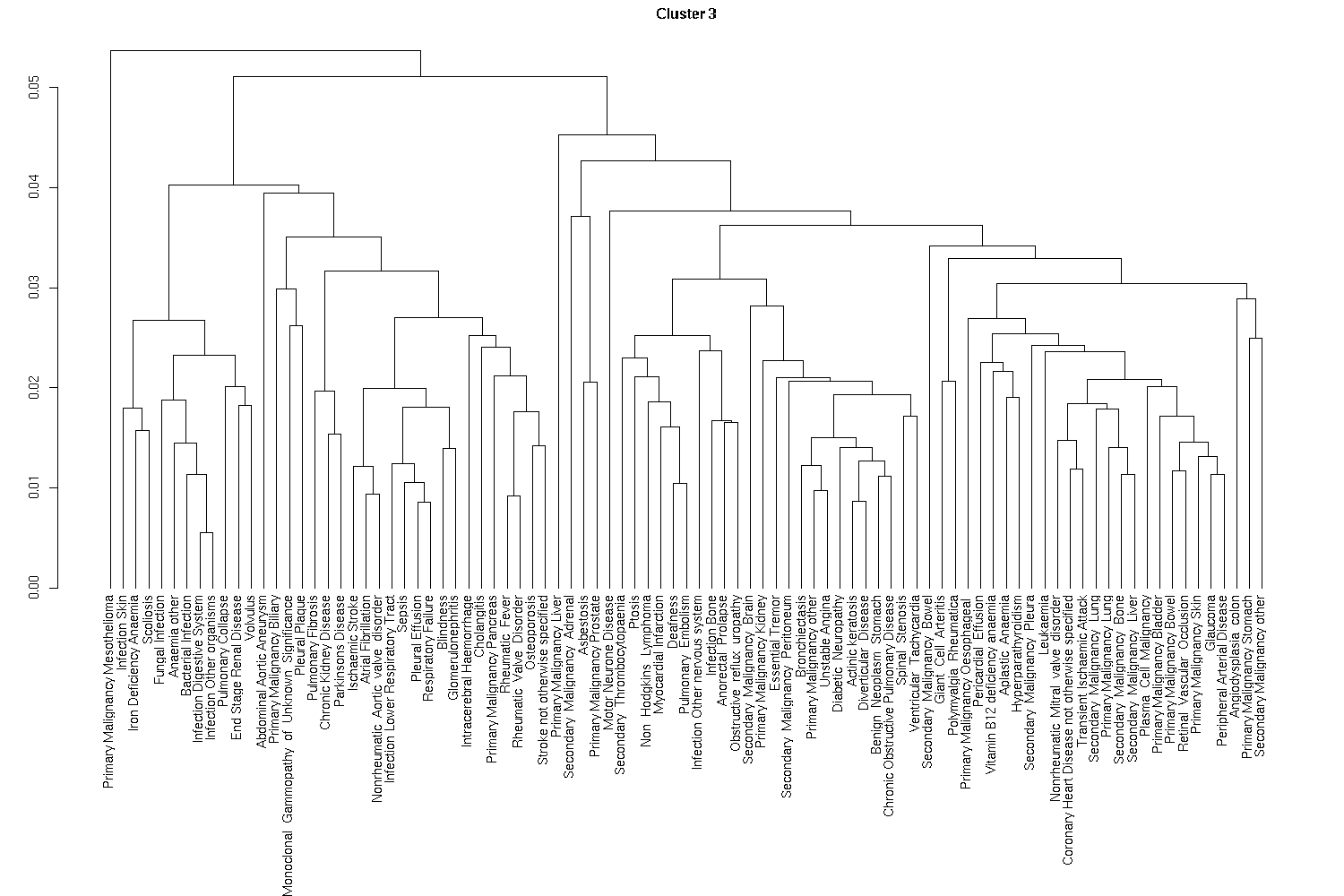


Supplementary Figure S14. Subdendrogram showing the result of the hierarchical agglomerative clustering algorithm for Cluster 4.


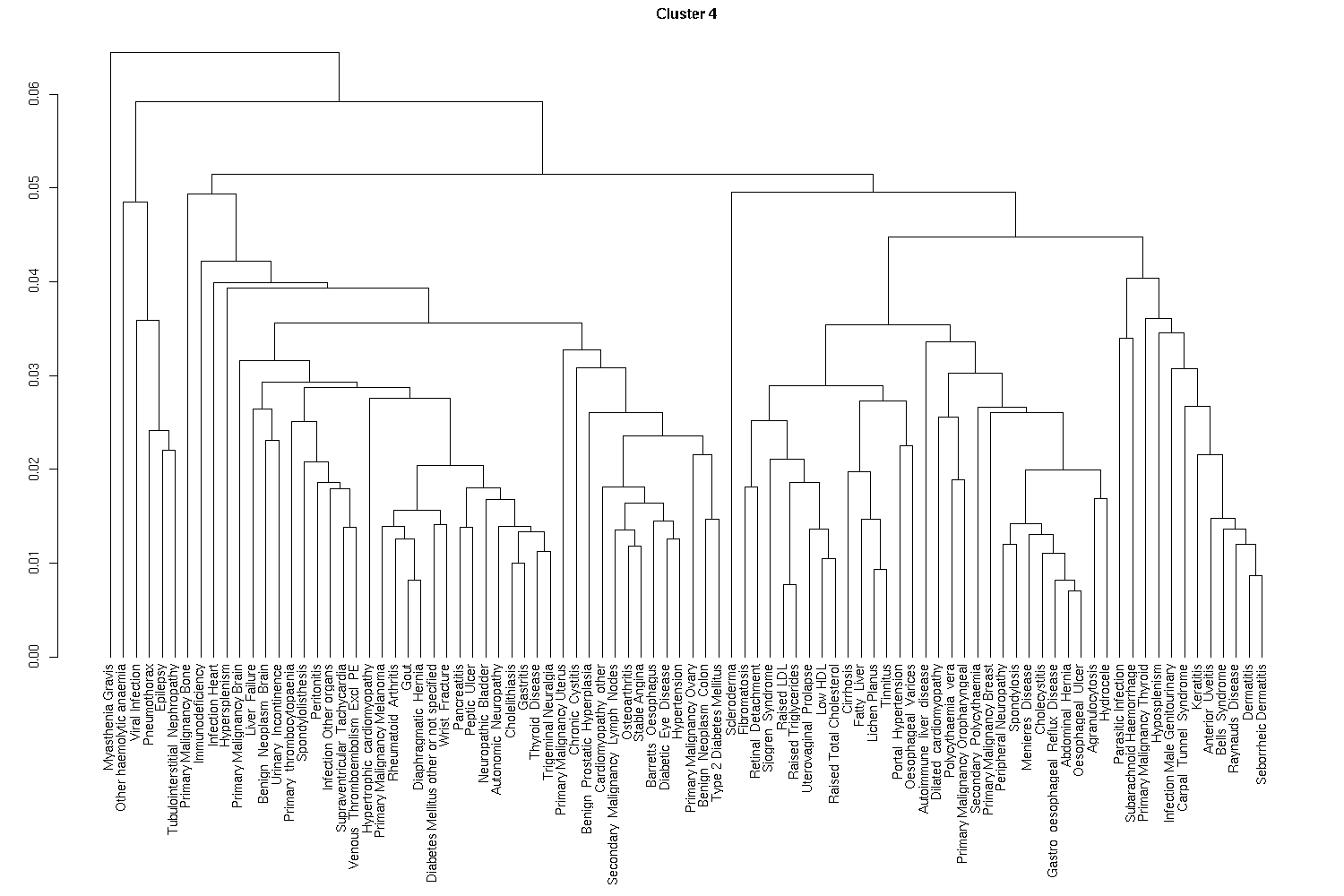


Supplementary Figure S15. Subdendrogram showing the result of the hierarchical agglomerative clustering algorithm for Cluster 5.


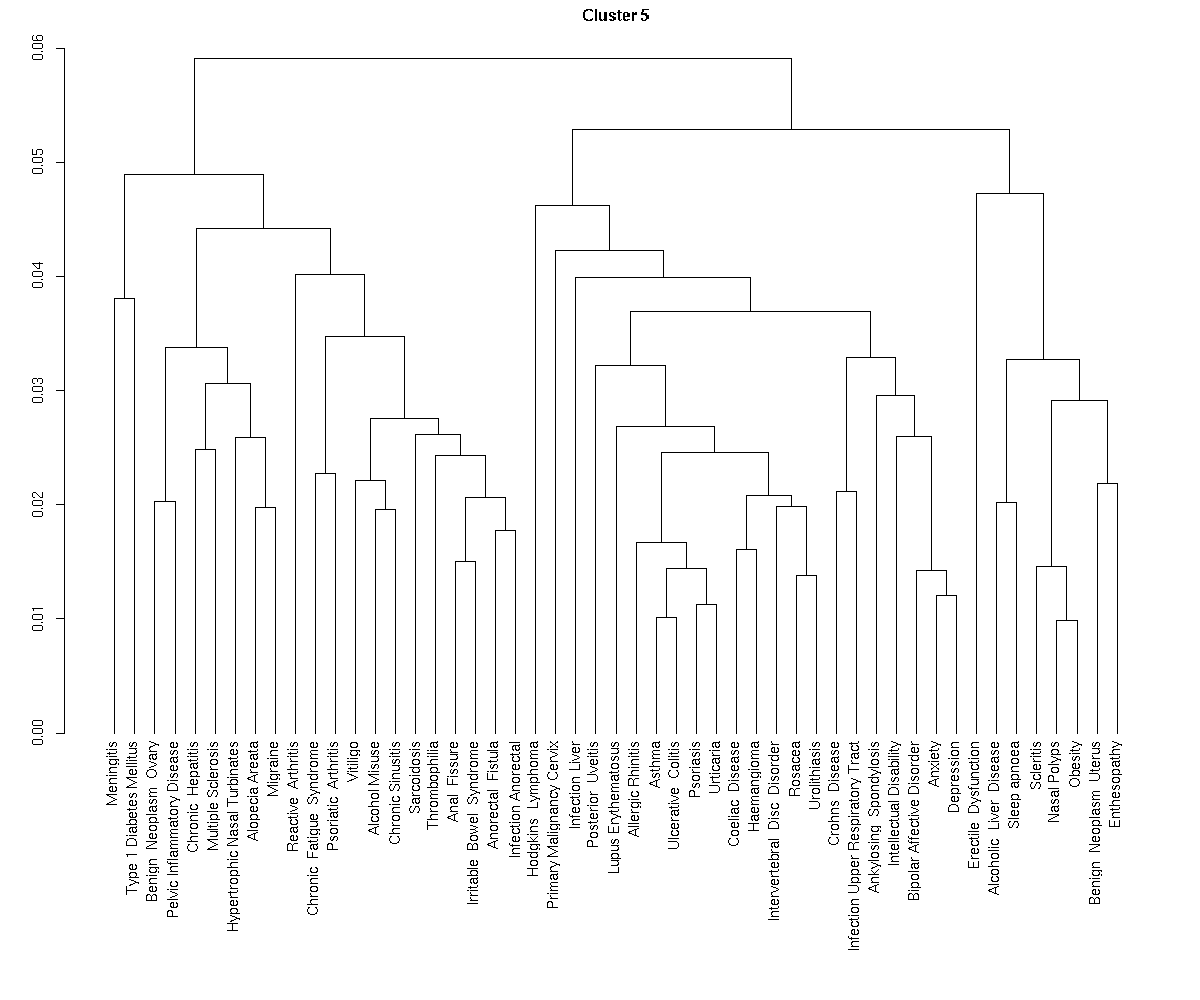


Supplementary Figure S16. Subdendrogram showing the result of the hierarchical agglomerative clustering algorithm for Cluster 6.


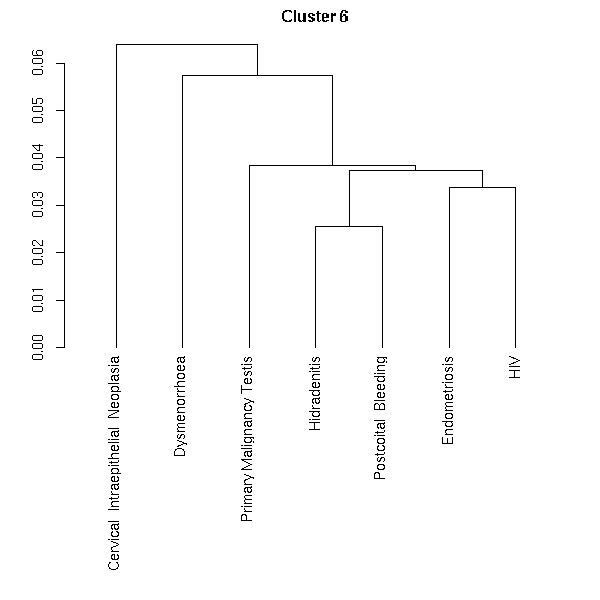


Supplementary Figure S17. Subdendrogram showing the result of the hierarchical agglomerative clustering algorithm for Cluster 7


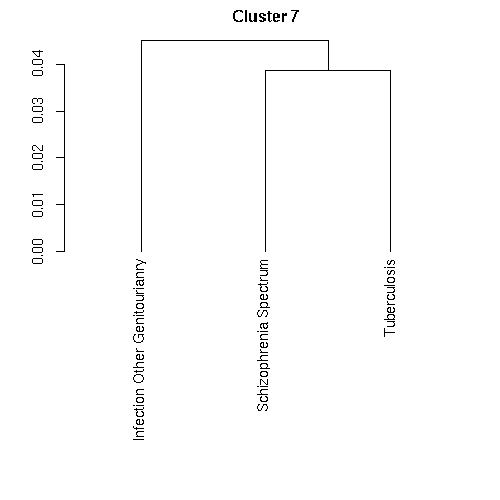


Supplementary Figure S18. Subdendrogram showing the result of the hierarchical agglomerative clustering algorithm for Cluster 8


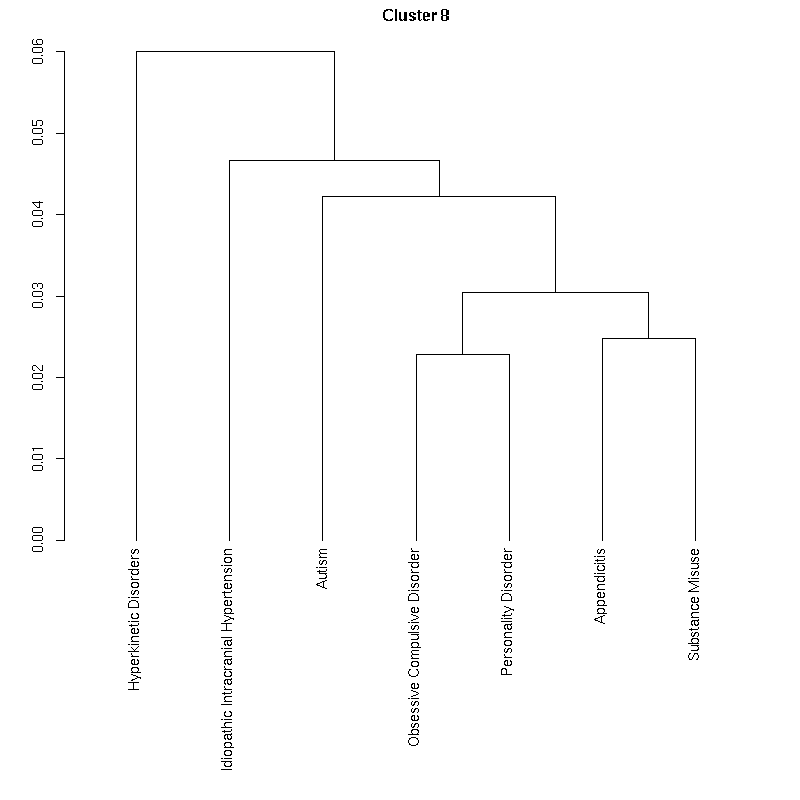


Supplementary Figure S19. Subdendrogram showing the result of the hierarchical agglomerative clustering algorithm for Cluster 9


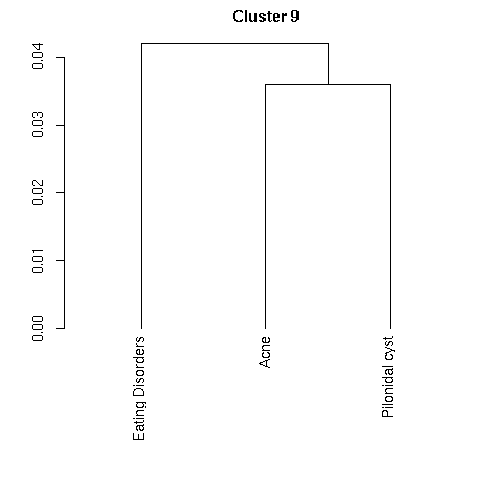


Supplementary Table S1. 289 diseases with their disease categories, rate of onset curve cluster, median (interquartile range (IQR)) age of first recorded diagnosis above 20 years, age at maximum rate of first recorded diagnosis above 20 years, β (coefficient of the age term) for the Gompertz model, adjusted R^2^ of the Gompertz-Makeham (GM) model and number of cases (n) on which the analyses were based. Diseases are listed in alphabetical order within their disease categories.

| Disease | Category | Main or outlier cluster | Median (IQR) age of first recorded diagnosis | Age at maximum rate of first recorded diagnosis | β of Gompertz model | Adjusted R^2^ of GM model | Number of cases (n) |
| --- | --- | --- | --- | --- | --- | --- | --- |
| Benign Neoplasm – Brain | Benign Neoplasm | Cluster 4 | 56 (42,70) | 83 | 0.050883604 | 0.984115766 | 7867 |
| Benign Neoplasm – Colon | Benign Neoplasm | Cluster 4 | 63 (52,72) | 79 | 0.081444826 | 0.994528701 | 90001 |
| Benign Neoplasm – Ovary | Benign Neoplasm | Cluster 5 | 38 (30,48) | 46 | -0.00524622 | 0.107482068 | 63973 |
| Benign Neoplasm – Stomach | Benign Neoplasm | Cluster 3 | 66 (56,75) | 84 | 0.098475726 | 0.994955138 | 16928 |
| Benign Neoplasm – Uterus | Benign Neoplasm | Cluster 5 | 52 (44,61) | 55 | 0.054186818 | 0.963334483 | 30934 |
| Cervical Intra-epithelial Neoplasia | Benign Neoplasm | Cluster 6 | 32 (27,40) | 26 | -0.055203059 | 0.927212686 | 44727 |
| Haemangioma | Benign Neoplasm | Cluster 5 | 48 (36,61) | 77 | 0.027967843 | 0.979141274 | 29871 |
| Leiomyoma | Benign Neoplasm | Outlier 5 | 46 (40,51) | 47 | 0.01847407 | 0.712846212 | 69937 |
| Hodgkins Lymphoma | Cancers | Cluster 5 | 39 (28,57) | 77 | 0.016191496 | 0.738461864 | 2114 |
| Leukaemia | Cancers | Cluster 3 | 67 (57,77) | 82 | 0.084880306 | 0.982667079 | 5845 |
| Monoclonal Gammopathy of Unknown Significance | Cancers | Cluster 3 | 72 (63,80) | 83 | 0.127516598 | 0.977646731 | 3398 |
| Myelodysplastic Syndrome | Cancers | Cluster 2 | 77 (67,84) | 83 | 0.103119764 | 0.98300671 | 2749 |
| Non Hodgkins Lymphoma | Cancers | Cluster 3 | 63 (51,74) | 84 | 0.07111727 | 0.993746787 | 8747 |
| Plasma Cell Malignancy | Cancers | Cluster 3 | 70 (61,79) | 82 | 0.109254667 | 0.987612225 | 2849 |
| Polycythaemia vera | Cancers | Cluster 4 | 59 (48,69) | 84 | 0.064598825 | 0.985084711 | 3516 |
| Primary Malignancy – Biliary | Cancers | Cluster 3 | 72 (63,81) | 83 | 0.113101572 | 0.968639204 | 1080 |
| Primary Malignancy – Bladder | Cancers | Cluster 3 | 71 (62,78) | 84 | 0.119057566 | 0.994580622 | 12346 |
| Primary Malignancy – Bone | Cancers | Cluster 4 | 59 (40,72) | 80 | 0.04767902 | 0.924565456 | 980 |
| Primary Malignancy – Bowel | Cancers | Cluster 3 | 69 (60,78) | 84 | 0.115916567 | 0.993954947 | 22635 |
| Primary Malignancy – Brain | Cancers | Cluster 4 | 59 (44,71) | 84 | 0.053855936 | 0.976062715 | 2543 |
| Primary Malignancy – Breast | Cancers | Cluster 4 | 59 (50,68) | 84 | 0.09236117 | 0.979204694 | 45415 |
| Primary Malignancy – Cervix | Cancers | Cluster 5 | 41 (34,54) | 82 | 0.01948834 | 0.410269487 | 3360 |
| Primary Malignancy – Kidney | Cancers | Cluster 3 | 68 (58,77) | 78 | 0.108875579 | 0.983540629 | 4907 |
| Primary Malignancy – Liver | Cancers | Cluster 3 | 70 (60,78) | 84 | 0.104038329 | 0.97632689 | 1203 |
| Primary Malignancy – Lung | Cancers | Cluster 3 | 71 (64,79) | 83 | 0.132382303 | 0.987443345 | 12306 |
| Primary Malignancy – Melanoma | Cancers | Cluster 4 | 58 (44,70) | 83 | 0.055612292 | 0.991926044 | 16061 |
| Primary Malignancy – Mesothelioma | Cancers | Cluster 3 | 72 (65,79) | 84 | 0.118725712 | 0.94419528 | 808 |
| Primary Malignancy – Multiple Sites | Cancers | Outlier 1 | 74 (66,83) | 83 | 0.099186877 | 0.955695468 | 386 |
| Primary Malignancy – Oesophageal | Cancers | Cluster 3 | 70 (62,79) | 82 | 0.132154707 | 0.973949388 | 3486 |
| Primary Malignancy – Oropharyngeal | Cancers | Cluster 4 | 61 (52,71) | 83 | 0.081057411 | 0.980817498 | 5171 |
| Primary Malignancy – other | Cancers | Cluster 3 | 66 (54,76) | 84 | 0.080678808 | 0.993573157 | 9335 |
| Primary Malignancy – Ovary | Cancers | Cluster 4 | 61 (50,71) | 84 | 0.071700912 | 0.984666668 | 4650 |
| Primary Malignancy – Pancreas | Cancers | Cluster 3 | 72 (63,81) | 84 | 0.120521103 | 0.983411373 | 2666 |
| Primary Malignancy – Prostate | Cancers | Cluster 3 | 71 (64,77) | 84 | 0.175954153 | 0.946572118 | 26758 |
| Primary Malignancy – Skin | Cancers | Cluster 3 | 68 (57,77) | 84 | 0.104789502 | 0.997114079 | 89308 |
| Primary Malignancy – Stomach | Cancers | Cluster 3 | 72 (63,80) | 84 | 0.116190487 | 0.982129687 | 2975 |
| Primary Malignancy – Testis | Cancers | Cluster 6 | 35 (29,43) | 32 | -0.025443803 | 0.572015471 | 2852 |
| Primary Malignancy – Thyroid | Cancers | Cluster 4 | 48 (37,62) | 83 | 0.03083709 | 0.857759458 | 2188 |
| Primary Malignancy – Uterus | Cancers | Cluster 4 | 64 (57,72) | 76 | 0.116489078 | 0.978336018 | 5971 |
| Secondary Malignancy – Adrenal | Cancers | Cluster 3 | 69 (61,77) | 82 | 0.114225465 | 0.964004964 | 1369 |
| Secondary Malignancy – Bone | Cancers | Cluster 3 | 71 (62,80) | 83 | 0.121396907 | 0.992945373 | 9499 |
| Secondary Malignancy – Bowel | Cancers | Cluster 3 | 68 (60,77) | 84 | 0.098079321 | 0.946397574 | 914 |
| Secondary Malignancy – Brain | Cancers | Cluster 3 | 67 (58,76) | 83 | 0.105643306 | 0.97743048 | 3271 |
| Secondary Malignancy – Liver | Cancers | Cluster 3 | 70 (61,79) | 83 | 0.116250776 | 0.991121291 | 11104 |
| Secondary Malignancy – Lung | Cancers | Cluster 3 | 70 (60,79) | 83 | 0.104271849 | 0.988998726 | 7731 |
| Secondary Malignancy – Lymph Nodes | Cancers | Cluster 4 | 64 (53,73) | 79 | 0.09092977 | 0.994944622 | 20149 |
| Secondary Malignancy – other | Cancers | Cluster 3 | 70 (60,80) | 84 | 0.103385591 | 0.988544739 | 4867 |
| Secondary Malignancy – Peritoneum | Cancers | Cluster 3 | 68 (59,76) | 82 | 0.104466071 | 0.983195373 | 4484 |
| Secondary Malignancy – Pleura | Cancers | Cluster 3 | 71 (61,79) | 83 | 0.108692839 | 0.972051537 | 2017 |
| Abdominal Aortic Aneurysm | Cardiovascular | Cluster 3 | 74 (66,81) | 83 | 0.134230919 | 0.968077428 | 13616 |
| Atrial Fibrillation | Cardiovascular | Cluster 3 | 74 (65,82) | 84 | 0.11856379 | 0.998185648 | 111515 |
| Atrioventricular Block, first degree | Cardiovascular | Cluster 2 | 80 (71,86) | 84 | 0.111951431 | 0.993427674 | 7321 |
| Atrioventricular Block, second degree | Cardiovascular | Cluster 2 | 76 (66,83) | 83 | 0.099931374 | 0.982491881 | 3189 |
| Atrioventricular Block, third degree | Cardiovascular | Cluster 2 | 77.5 (68,85) | 84 | 0.114491646 | 0.984560035 | 4580 |
| Bifascicular Block | Cardiovascular | Cluster 1 | 82 (73,87) | 84 | 0.109320099 | 0.965358714 | 655 |
| Cardiomyopathy – other | Cardiovascular | Cluster 4 | 62 (51,73) | 77 | 0.072584627 | 0.994013337 | 7724 |
| Coronary Heart Disease (not otherwise specified) | Cardiovascular | Cluster 3 | 70 (60,79) | 84 | 0.116150968 | 0.992593314 | 29569 |
| Dilated cardiomyopathy | Cardiovascular | Cluster 4 | 61 (50,71) | 83 | 0.074409757 | 0.986380242 | 4493 |
| Heart Failure | Cardiovascular | Cluster 2 | 76 (66,84) | 84 | 0.12771274 | 0.99755392 | 67132 |
| Hypertension | Cardiovascular | Cluster 4 | 59 (50,69) | 84 | 0.089721124 | 0.998870104 | 649706 |
| Hypertrophic cardiomyopathy | Cardiovascular | Cluster 4 | 59 (47,71) | 81 | 0.06256945 | 0.96112744 | 1701 |
| Intracerebral Haemorrhage | Cardiovascular | Cluster 3 | 71 (58,81) | 84 | 0.089022997 | 0.99500196 | 8235 |
| Ischaemic Stroke | Cardiovascular | Cluster 3 | 74 (63,83) | 84 | 0.113123217 | 0.997812044 | 34227 |
| Left Bundle Branch Block | Cardiovascular | Cluster 2 | 77 (67,85) | 84 | 0.124420924 | 0.994085597 | 12868 |
| Multiple valve disorder | Cardiovascular | Cluster 2 | 77 (67,84) | 84 | 0.111973686 | 0.994289531 | 13749 |
| Myocardial Infarction | Cardiovascular | Cluster 3 | 65 (55,76) | 84 | 0.113527315 | 0.990992485 | 83674 |
| Non-rheumatic Aortic valve disorder | Cardiovascular | Cluster 3 | 75 (65,82) | 84 | 0.104076573 | 0.993300737 | 24568 |
| Non-rheumatic Mitral valve disorder | Cardiovascular | Cluster 3 | 69 (56,79) | 84 | 0.078242523 | 0.994067043 | 23196 |
| Pericardial Effusion | Cardiovascular | Cluster 3 | 67 (54,78) | 84 | 0.078445378 | 0.990374481 | 4600 |
| Peripheral Arterial Disease | Cardiovascular | Cluster 3 | 68 (59,77) | 83 | 0.108430695 | 0.988827853 | 45443 |
| Primary Pulmonary Hypertension | Cardiovascular | Cluster 2 | 75 (64,83) | 83 | 0.099711745 | 0.990992891 | 4697 |
| Pulmonary Embolism | Cardiovascular | Cluster 3 | 63 (47,75) | 84 | 0.061623089 | 0.995628756 | 29687 |
| Raynauds Disease | Cardiovascular | Cluster 4 | 48 (36,63) | 83 | 0.031121873 | 0.987001211 | 30885 |
| Rheumatic Valve Disorder | Cardiovascular | Cluster 3 | 71 (57,81) | 84 | 0.077586627 | 0.987993474 | 5735 |
| Right Bundle Branch Block | Cardiovascular | Cluster 2 | 75.5 (62,84) | 84 | 0.086367796 | 0.995528139 | 12803 |
| Secondary Pulmonary Hypertension | Cardiovascular | Cluster 2 | 77 (66,84) | 84 | 0.106087698 | 0.981787262 | 2698 |
| Sick Sinus Syndrome | Cardiovascular | Cluster 2 | 75 (66,83) | 83 | 0.100817736 | 0.982537801 | 3277 |
| Stable Angina | Cardiovascular | Cluster 4 | 64 (55,73) | 84 | 0.110757178 | 0.995639205 | 135647 |
| Stroke – not otherwise specified | Cardiovascular | Cluster 3 | 72 (61,81) | 84 | 0.108998037 | 0.99615606 | 29979 |
| Subarachnoid Haemorrhage | Cardiovascular | Cluster 4 | 52 (41,65) | 84 | 0.043229714 | 0.95392155 | 6369 |
| Subdural haematoma | Cardiovascular | Cluster 2 | 77 (64,85) | 84 | 0.092604127 | 0.991167221 | 2652 |
| Supraventricular Tachycardia | Cardiovascular | Cluster 4 | 59 (44,72) | 81 | 0.055114921 | 0.995768105 | 22373 |
| Transient Ischaemic Attack | Cardiovascular | Cluster 3 | 70 (61,79) | 84 | 0.12162865 | 0.997373146 | 53154 |
| Trifascicular Block | Cardiovascular | Cluster 1 | 82 (76,87) | 84 | 0.118130186 | 0.961058933 | 837 |
| Unstable Angina | Cardiovascular | Cluster 3 | 66 (57,76) | 84 | 0.123206743 | 0.99144951 | 45761 |
| Venous thromboembolism (Excluding Pulmonary Embolism) | Cardiovascular | Cluster 4 | 59 (42,72) | 84 | 0.053043028 | 0.995958443 | 46710 |
| Ventricular Tachycardia | Cardiovascular | Cluster 3 | 67 (55,77) | 83 | 0.082201703 | 0.990065601 | 5678 |
| Abdominal Hernia | Digestive | Cluster 4 | 53 (40,65) | 84 | 0.044787122 | 0.999168791 | 192837 |
| Alcoholic Liver Disease | Digestive | Cluster 5 | 52 (43,61) | 63 | 0.041207068 | 0.97680353 | 11425 |
| Anal Fissure | Digestive | Cluster 5 | 38 (30,50) | 46 | -0.00362564 | 0.742850636 | 71525 |
| Angiodysplasia of colon | Digestive | Cluster 3 | 71 (61,79) | 81 | 0.102176516 | 0.970070567 | 2420 |
| Anorectal Fistula | Digestive | Cluster 5 | 42 (33,52) | 51 | 0.003268171 | 0.729380289 | 14891 |
| Anorectal Prolapse | Digestive | Cluster 3 | 64 (50,77) | 84 | 0.070740458 | 0.991077782 | 8869 |
| Appendicitis | Digestive | Cluster 8 | 31 (25,42) | 21 | -0.023356409 | 0.98380916 | 92820 |
| Autoimmune liver disease | Digestive | Cluster 4 | 59 (48,68) | 75 | 0.061935645 | 0.966236551 | 2198 |
| Barrett's Oesophagus | Digestive | Cluster 4 | 64 (54,74) | 83 | 0.091500063 | 0.992508075 | 16042 |
| Cholangitis | Digestive | Cluster 3 | 71 (57,81) | 84 | 0.081840897 | 0.99170425 | 4958 |
| Cholecystitis | Digestive | Cluster 4 | 54 (40,67) | 82 | 0.044519065 | 0.993556105 | 56065 |
| Cholelithiasis | Digestive | Cluster 4 | 55 (42,69) | 84 | 0.049629265 | 0.995280472 | 110010 |
| Cirrhosis | Digestive | Cluster 4 | 55 (45,64) | 82 | 0.057536531 | 0.980743952 | 11153 |
| Coeliac Disease | Digestive | Cluster 5 | 48 (36,62) | 77 | 0.028626001 | 0.973837728 | 9504 |
| Crohns Disease | Digestive | Cluster 5 | 40 (29,55) | 84 | 0.010546717 | 0.829676863 | 12604 |
| Diaphragmatic Hernia | Digestive | Cluster 4 | 60 (48,71) | 84 | 0.068605542 | 0.999107617 | 155973 |
| Diverticular Disease | Digestive | Cluster 3 | 67 (57,75) | 84 | 0.116994119 | 0.998669007 | 139773 |
| Fatty Liver | Digestive | Cluster 4 | 54 (44,64) | 69 | 0.050090519 | 0.993524252 | 13395 |
| Gastritis | Digestive | Cluster 4 | 54 (40,67) | 82 | 0.045155795 | 0.998484264 | 197361 |
| Gastro-oesophageal Reflux Disease | Digestive | Cluster 4 | 53 (40,65) | 84 | 0.044388136 | 0.996677879 | 287374 |
| Irritable Bowel Syndrome | Digestive | Cluster 5 | 40 (30,52) | 66 | 0.001075635 | 0.888007687 | 199449 |
| Liver Failure | Digestive | Cluster 4 | 59 (47,70) | 84 | 0.060922887 | 0.976590064 | 4373 |
| Oesophageal Ulcer | Digestive | Cluster 4 | 55 (42,67) | 84 | 0.049724722 | 0.997997852 | 184269 |
| Oesophageal Varices | Digestive | Cluster 4 | 57 (47,67) | 76 | 0.067390591 | 0.983633817 | 4319 |
| Pancreatitis | Digestive | Cluster 4 | 54 (40,68) | 84 | 0.04410926 | 0.990808723 | 18938 |
| Peptic Ulcer | Digestive | Cluster 4 | 53 (38,68) | 84 | 0.042396915 | 0.995488339 | 73465 |
| Peritonitis | Digestive | Cluster 4 | 57 (41,72) | 84 | 0.049131558 | 0.995623614 | 18267 |
| Portal Hypertension | Digestive | Cluster 4 | 57 (47,66) | 82 | 0.064486579 | 0.984130567 | 3996 |
| Ulcerative Colitis | Digestive | Cluster 5 | 43 (32,59) | 79 | 0.018853521 | 0.931213225 | 20981 |
| Volvulus | Digestive | Cluster 3 | 67 (53,79) | 84 | 0.072709551 | 0.981025701 | 3251 |
| Deafness | Ear | Cluster 3 | 61 (46,74) | 84 | 0.063335421 | 0.998990314 | 218521 |
| Meniere's Disease | Ear | Cluster 4 | 56 (44,67) | 82 | 0.056136699 | 0.985638988 | 11755 |
| Tinnitus | Ear | Cluster 4 | 54 (43,65) | 69 | 0.045362253 | 0.99496919 | 103594 |
| Diabetes Mellitus – other or not specified | Endocrine | Cluster 4 | 58 (43,71) | 83 | 0.054824485 | 0.986764084 | 10722 |
| Hyperparathyroidism | Endocrine | Cluster 3 | 67 (54,78) | 83 | 0.083181977 | 0.990479227 | 6484 |
| Low high density lipoprotein-cholesterol | Endocrine | Cluster 4 | 57 (46,67) | 73 | 0.067673635 | 0.994654134 | 218427 |
| Obesity | Endocrine | Cluster 5 | 46 (35,58) | 65 | 0.020353594 | 0.967107165 | 573815 |
| Polycystic Ovarian Syndrome | Endocrine | Outlier 9 | 29 (25,33) | 27 | -0.084315796 | 0.91297945 | 15856 |
| Raised low density lipoprotein-cholesterol | Endocrine | Cluster 4 | 57 (48,66) | 71 | 0.078991462 | 0.997540566 | 502613 |
| Raised Total Cholesterol | Endocrine | Cluster 4 | 56 (47,65) | 71 | 0.078506618 | 0.997271225 | 786637 |
| Raised Triglycerides | Endocrine | Cluster 4 | 57 (48,66) | 69 | 0.076149651 | 0.998326713 | 365716 |
| Syndrome of Inappropriate AntiDiuretic Hormone | Endocrine | Cluster 2 | 79 (69,85) | 84 | 0.107743446 | 0.980993254 | 1332 |
| Thyroid Disease | Endocrine | Cluster 4 | 53 (41,67) | 84 | 0.046916187 | 0.99139503 | 166754 |
| Type 1 Diabetes Mellitus | Endocrine | Cluster 5 | 36 (28,48) | 36 | -0.007998467 | 0.531074155 | 8880 |
| Type 2 Diabetes Mellitus | Endocrine | Cluster 4 | 61 (51,70) | 78 | 0.087672376 | 0.998634241 | 186940 |
| Anterior Uveitis | Eye | Cluster 4 | 47 (35,62) | 84 | 0.029775541 | 0.96178507 | 22376 |
| Blindness | Eye | Cluster 3 | 72 (54,83) | 84 | 0.072346462 | 0.996658793 | 29403 |
| Cataract | Eye | Cluster 2 | 74 (66,81) | 84 | 0.127495003 | 0.996792395 | 189350 |
| Diabetic Eye Disease | Eye | Cluster 4 | 64 (53,73) | 82 | 0.080678836 | 0.994690347 | 61859 |
| Glaucoma | Eye | Cluster 3 | 68 (58,77) | 83 | 0.107114633 | 0.997830856 | 53546 |
| Keratitis | Eye | Cluster 4 | 48 (34,63) | 82 | 0.029677064 | 0.977447705 | 15552 |
| Macular Degeneration | Eye | Cluster 2 | 77 (69,84) | 84 | 0.12408693 | 0.99687634 | 36868 |
| Posterior Uveitis | Eye | Cluster 5 | 45 (33,58) | 80 | 0.020455979 | 0.784555429 | 1638 |
| Ptosis | Eye | Cluster 3 | 63 (49,74) | 84 | 0.065473258 | 0.990979243 | 9972 |
| Retinal Detachment | Eye | Cluster 4 | 59 (47,67) | 67 | 0.054160869 | 0.936857844 | 17676 |
| Retinal Vascular Occlusion | Eye | Cluster 3 | 70 (60,78) | 82 | 0.107697226 | 0.991202791 | 12069 |
| Scleritis | Eye | Cluster 5 | 47 (38,59) | 66 | 0.024055219 | 0.974255573 | 14404 |
| Acute Kidney Injury | Genitourinary | Cluster 2 | 79 (68,87) | 84 | 0.103756755 | 0.998450287 | 42710 |
| Benign Prostatic Hyperplasia | Genitourinary | Cluster 4 | 66 (59,74) | 81 | 0.140395098 | 0.988174484 | 103740 |
| Chronic Cystitis | Genitourinary | Cluster 4 | 61 (43,72) | 82 | 0.055285092 | 0.973177747 | 6358 |
| Chronic Kidney Disease | Genitourinary | Cluster 3 | 74 (68,81) | 84 | 0.153536717 | 0.991548857 | 117429 |
| Dysmenorrhoea | Genitourinary | Cluster 6 | 35 (28,42) | 42 | -0.114585844 | 0.862159114 | 70356 |
| End Stage Renal Disease | Genitourinary | Cluster 3 | 66 (49,78) | 84 | 0.06594187 | 0.992838725 | 7826 |
| Endometrial Hyperplasia | Genitourinary | Outlier 5 | 48 (41,55) | 49 | 0.028995366 | 0.85021588 | 12351 |
| Endometriosis | Genitourinary | Cluster 6 | 36 (30,43) | 37 | -0.042422471 | 0.792462394 | 41133 |
| Erectile Dysfunction | Genitourinary | Cluster 5 | 56 (47,64) | 66 | 0.0508924 | 0.962651498 | 147079 |
| Female Infertility | Genitourinary | Outlier 8 | 31 (28,36) | 31 | -0.118208621 | 0.792987293 | 47194 |
| Glomerulonephritis | Genitourinary | Cluster 3 | 71 (53,82) | 84 | 0.069983614 | 0.994256023 | 16227 |
| Hydrocele | Genitourinary | Cluster 4 | 55 (40,67) | 82 | 0.042987559 | 0.953092844 | 17553 |
| Male infertility | Genitourinary | Outlier 8 | 34 (30,38) | 34 | -0.081172552 | 0.737750054 | 12027 |
| Menorrhagia | Genitourinary | Outlier 6 | 41 (34,46) | 45 | -0.09705905 | 0.825306345 | 220699 |
| Neuropathic Bladder | Genitourinary | Cluster 4 | 57 (45,70) | 84 | 0.056784548 | 0.997183183 | 30181 |
| Obstructive and reflux uropathy | Genitourinary | Cluster 3 | 61 (43,75) | 84 | 0.054113068 | 0.99590789 | 20985 |
| Postcoital Bleeding | Genitourinary | Cluster 6 | 35 (28,43) | 36 | -0.068367737 | 0.984084305 | 45617 |
| Postmenopausal Bleeding | Genitourinary | Outlier 3 | 57 (53,64) | 56 | 0.133130929 | 0.930584662 | 64575 |
| Tubulo-interstitial Nephropathy | Genitourinary | Cluster 4 | 40 (28,59) | 84 | 0.018052674 | 0.953411958 | 9637 |
| Urinary Incontinence | Genitourinary | Cluster 4 | 55 (43,70) | 84 | 0.057223849 | 0.952634436 | 119475 |
| Urolithiasis | Genitourinary | Cluster 5 | 46 (35,58) | 79 | 0.021703056 | 0.970470572 | 77048 |
| Uterovaginal Prolapse | Genitourinary | Cluster 4 | 57 (46,67) | 76 | 0.068104464 | 0.980883369 | 91077 |
| Agranulocytosis | Haematological/ Immuunoligcal | Cluster 4 | 58 (44,69) | 75 | 0.052896776 | 0.986685566 | 17567 |
| Anaemia – other | Haematological/ Immuunoligcal | Cluster 3 | 61 (40,77) | 84 | 0.052039602 | 0.966466263 | 143637 |
| Aplastic Anaemia | Haematological/ Immuunoligcal | Cluster 3 | 67 (53,79) | 83 | 0.074029153 | 0.985881738 | 3137 |
| Folate Deficiency Anaemia | Haematological/ Immuunoligcal | Cluster 2 | 72 (53,84) | 83 | 0.071900714 | 0.976190174 | 4055 |
| Hypersplenism | Haematological/ Immuunoligcal | Cluster 4 | 54 (40,68) | 83 | 0.043781086 | 0.962065901 | 4074 |
| Hyposplenism | Haematological/ Immuunoligcal | Cluster 4 | 49 (32,65) | 76 | 0.030805159 | 0.880189315 | 3529 |
| Immunodeficiency | Haematological/ Immuunoligcal | Cluster 4 | 57 (40,70) | 84 | 0.048329057 | 0.951745907 | 1319 |
| Iron Deficiency Anaemia | Haematological/ Immuunoligcal | Cluster 3 | 52 (38,74) | 84 | 0.044762347 | 0.905228625 | 124499 |
| Other haemolytic anaemia | Haematological/ Immuunoligcal | Cluster 4 | 47 (32,68) | 83 | 0.032957581 | 0.90611428 | 2554 |
| Primary thrombocytopaenia | Haematological/ Immuunoligcal | Cluster 4 | 56 (37,71) | 83 | 0.044338975 | 0.968001002 | 3606 |
| Sarcoidosis | Haematological/ Immuunoligcal | Cluster 5 | 40 (32,51) | 50 | 0.001136233 | 0.516379367 | 6602 |
| Secondary Polycythaemia | Haematological/ Immuunoligcal | Cluster 4 | 57 (46,68) | 78 | 0.058214313 | 0.977804667 | 3121 |
| Secondary Thrombocytopaenia | Haematological/ Immuunoligcal | Cluster 3 | 60 (41,74) | 83 | 0.053425319 | 0.972160751 | 12278 |
| Thrombophilia | Haematological/ Immuunoligcal | Cluster 5 | 40 (31,52) | 75 | 0.003921105 | 0.445241496 | 4006 |
| Vitamin B12 deficiency anaemia | Haematological/ Immuunoligcal | Cluster 3 | 65 (47,77) | 84 | 0.065871657 | 0.988269128 | 14961 |
| Bacterial Infection | Infections | Cluster 3 | 59 (39,77) | 84 | 0.048459924 | 0.989729302 | 267876 |
| Chronic Hepatitis | Infections | Cluster 5 | 39 (31,48) | 41 | -0.013635563 | 0.6905962 | 8559 |
| Encephalitis | Infections | Outlier 2 | 58 (40,72) | 84 | 0.048409732 | 0.910678895 | 657 |
| Fungal Infection | Infections | Cluster 3 | 64 (41,78) | 83 | 0.052867803 | 0.985307701 | 19795 |
| Human Immunodeficiency Virus (HIV) | Infections | Cluster 6 | 36 (30,44) | 40 | -0.036075172 | 0.805041697 | 3253 |
| Infection – Anorectal | Infections | Cluster 5 | 40 (31,51) | 43 | -0.001396292 | 0.603280204 | 11942 |
| Infection – Bone | Infections | Cluster 3 | 61 (45,75) | 84 | 0.059009945 | 0.991832545 | 7874 |
| Infection – Digestive System | Infections | Cluster 3 | 64 (45,79) | 84 | 0.056950435 | 0.99804433 | 55098 |
| Infection – Ear/Upper Respiratory Tract | Infections | Cluster 5 | 39 (29,56) | 84 | 0.01208242 | 0.946020875 | 43717 |
| Infection – Eye | Infections | Cluster 2 | 71 (49,84) | 84 | 0.061729829 | 0.977822204 | 3373 |
| Infection – Heart | Infections | Cluster 4 | 57 (40,71) | 84 | 0.049069032 | 0.965862314 | 1717 |
| Infection – Liver | Infections | Cluster 5 | 44 (34,56) | 83 | 0.020603251 | 0.665052071 | 7211 |
| Infection – Lower Respiratory Tract | Infections | Cluster 3 | 71 (55,83) | 84 | 0.073475 | 0.998507373 | 136683 |
| Infection – Male Genitourinary | Infections | Cluster 4 | 48 (34,65) | 81 | 0.03239116 | 0.960722682 | 8225 |
| Infection – Other Genitourinary | Infections | Cluster 7 | 32 (26,43) | 83 | -0.005351913 | 0.752338243 | 7575 |
| Infection – Other nervous system | Infections | Cluster 3 | 62 (45,75) | 84 | 0.057834099 | 0.979893632 | 3067 |
| Infection – Other organisms | Infections | Cluster 3 | 64 (44,79) | 84 | 0.056615217 | 0.999077998 | 188352 |
| Infection – Other organs | Infections | Cluster 4 | 53 (35,70) | 83 | 0.039778141 | 0.988267585 | 70123 |
| Infection – Skin | Infections | Cluster 3 | 57 (39,75) | 84 | 0.044769119 | 0.993346427 | 78012 |
| Meningitis | Infections | Cluster 5 | 36 (28,48) | 38 | -0.002397908 | 0.091579163 | 2745 |
| Parasitic Infection | Infections | Cluster 4 | 47 (35,63) | 84 | 0.027010961 | 0.880210932 | 2922 |
| Pelvic Inflammatory Disease | Infections | Cluster 5 | 38 (31,47) | 42 | -0.009551274 | 0.349621404 | 32843 |
| Rheumatic Fever | Infections | Cluster 3 | 69 (52,80) | 84 | 0.067181522 | 0.984793364 | 6497 |
| Septicaemia | Infections | Cluster 3 | 72 (58,82) | 84 | 0.080598285 | 0.998110037 | 27259 |
| Tuberculosis | Infections | Cluster 7 | 33 (26,46) | 83 | -0.00120363 | 0.828449961 | 14090 |
| Urinary Tract Infection | Infections | Cluster 2 | 75 (56,84) | 84 | 0.069619069 | 0.999166886 | 103251 |
| Viral Infection | Infections | Cluster 4 | 44 (31,65) | 84 | 0.025799309 | 0.964129628 | 42174 |
| Ankylosing Spondylosis | Musculoskeletal | Cluster 5 | 39 (30,53) | 82 | 0.007549223 | 0.33834975 | 6034 |
| Carpal Tunnel Syndrome | Musculoskeletal | Cluster 4 | 50 (40,62) | 83 | 0.040885457 | 0.926256506 | 123312 |
| Collapsed Vertebra | Musculoskeletal | Cluster 2 | 75 (63,83) | 84 | 0.089523111 | 0.993390763 | 7785 |
| Enteropathic Arthropathy | Musculoskeletal | Outlier 4 | 50 (37,63) | 82 | 0.035776299 | 0.757141259 | 377 |
| Enthesopathy | Musculoskeletal | Cluster 5 | 49 (40,60) | 76 | 0.037928806 | 0.983182644 | 598066 |
| Fibromatosis | Musculoskeletal | Cluster 4 | 62 (53,69) | 67 | 0.088362383 | 0.992102211 | 34339 |
| Fracture – Hip | Musculoskeletal | Cluster 1 | 80 (70,87) | 84 | 0.097929893 | 0.995924429 | 27259 |
| Fracture – Wrist | Musculoskeletal | Cluster 4 | 57 (40,70) | 84 | 0.047176622 | 0.976946454 | 64922 |
| Giant Cell Arteritis | Musculoskeletal | Cluster 3 | 71 (63,78) | 82 | 0.119896916 | 0.983196153 | 5454 |
| Gout | Musculoskeletal | Cluster 4 | 59 (47,71) | 82 | 0.073072673 | 0.979134054 | 97173 |
| Intervertebral Disc Disorder | Musculoskeletal | Cluster 5 | 47 (37,59) | 83 | 0.029840088 | 0.887134198 | 104352 |
| Juvenile Arthritis | Musculoskeletal | Outlier 7 | 34 (26,48) | 84 | 0.0058322 | 0.270055325 | 386 |
| Lupus Erythematosus | Musculoskeletal | Cluster 5 | 44 (34,57) | 82 | 0.018085026 | 0.87125319 | 5214 |
| Osteoarthritis | Musculoskeletal | Cluster 4 | 62 (53,71) | 84 | 0.097015335 | 0.998400899 | 397453 |
| Osteoporosis | Musculoskeletal | Cluster 3 | 71 (61,80) | 84 | 0.113594824 | 0.99084868 | 88750 |
| Polymyalgia Rheumatica | Musculoskeletal | Cluster 3 | 72 (65,78) | 84 | 0.138516977 | 0.986470227 | 26997 |
| Psoriatic Arthritis | Musculoskeletal | Cluster 5 | 47 (37,57) | 55 | 0.017572748 | 0.964353632 | 7952 |
| Reactive Arthritis | Musculoskeletal | Cluster 5 | 40 (30,51) | 82 | -0.000254522 | 0.153614261 | 3006 |
| Rheumatoid Arthritis | Musculoskeletal | Cluster 4 | 58 (46,71) | 84 | 0.060869432 | 0.997029197 | 35841 |
| Scleroderma | Musculoskeletal | Cluster 4 | 55 (44,66) | 83 | 0.047925693 | 0.922390396 | 1363 |
| Scoliosis | Musculoskeletal | Cluster 3 | 57 (37,76) | 84 | 0.044304898 | 0.989141975 | 16432 |
| Sjogren Syndrome | Musculoskeletal | Cluster 4 | 58 (48,67) | 75 | 0.064481486 | 0.986838431 | 3493 |
| Spinal Stenosis | Musculoskeletal | Cluster 3 | 67 (57,76) | 81 | 0.100491822 | 0.996773735 | 24129 |
| Spondylolisthesis | Musculoskeletal | Cluster 4 | 60 (45,72) | 83 | 0.060205987 | 0.992729593 | 10785 |
| Spondylosis | Musculoskeletal | Cluster 4 | 57 (47,68) | 84 | 0.073552999 | 0.989562724 | 143760 |
| Autonomic Neuropathy | Neurological | Cluster 4 | 56 (43,68) | 84 | 0.052335867 | 0.989975602 | 7731 |
| Bell's Palsy | Neurological | Cluster 4 | 46 (34,61) | 80 | 0.025559461 | 0.98041595 | 21985 |
| Chronic Fatigue Syndrome | Neurological | Cluster 5 | 44 (35,54) | 54 | 0.007330068 | 0.963551057 | 47025 |
| Diabetic Neuropathy | Neurological | Cluster 3 | 66 (56,76) | 80 | 0.097716778 | 0.993684657 | 12137 |
| Epilepsy | Neurological | Cluster 4 | 44 (31,62) | 84 | 0.024084259 | 0.988370972 | 36469 |
| Essential Tremor | Neurological | Cluster 3 | 66 (52,75) | 84 | 0.069606338 | 0.963506234 | 7416 |
| Idiopathic Intracranial Hypertension | Neurological | Cluster 8 | 32 (26,43) | 24 | -0.0185278 | 0.421580956 | 1455 |
| Migraine | Neurological | Cluster 5 | 38 (29,48) | 47 | -0.013190767 | 0.926265372 | 196126 |
| Motor Neurone Disease | Neurological | Cluster 3 | 68 (56,77) | 82 | 0.084526439 | 0.970501602 | 1273 |
| Multiple Sclerosis | Neurological | Cluster 5 | 41 (32,50) | 43 | -0.006687723 | 0.585234919 | 8855 |
| Myasthenia Gravis | Neurological | Cluster 4 | 60 (42,72) | 83 | 0.052432684 | 0.920618003 | 1253 |
| Parkinson's Disease | Neurological | Cluster 3 | 74 (66,81) | 84 | 0.138387521 | 0.99381883 | 12395 |
| Peripheral Neuropathy | Neurological | Cluster 4 | 56 (44,67) | 83 | 0.053888495 | 0.997919835 | 69126 |
| Trigeminal Neuralgia | Neurological | Cluster 4 | 55 (43,67) | 84 | 0.053599761 | 0.987110358 | 17861 |
| Alcohol Misuse | Psychiatric | Cluster 5 | 42 (32,54) | 66 | 0.007099737 | 0.891954693 | 99913 |
| Anxiety | Psychiatric | Cluster 5 | 40 (30,53) | 84 | 0.007660398 | 0.753713971 | 416825 |
| Autism | Psychiatric | Cluster 8 | 35 (26,45) | 21 | -0.018591911 | 0.567438053 | 2425 |
| Bipolar Affective Disorder | Psychiatric | Cluster 5 | 40 (31,52) | 84 | 0.0055679 | 0.387609874 | 15231 |
| Delirium | Psychiatric | Cluster 1 | 83 (75,89) | 84 | 0.107001743 | 0.994248173 | 10657 |
| Dementia | Psychiatric | Cluster 1 | 83 (77,88) | 84 | 0.161216647 | 0.991036804 | 40305 |
| Depression | Psychiatric | Cluster 5 | 39 (30,51) | 84 | 0.004861746 | 0.231609861 | 569690 |
| Eating Disorders | Psychiatric | Cluster 9 | 27 (23,34) | 21 | -0.040094994 | 0.877343792 | 6949 |
| Hyperkinetic Disorders | Psychiatric | Cluster 8 | 31 (24,40) | 21 | -0.026591853 | 0.661110426 | 1430 |
| Intellectual Disability | Psychiatric | Cluster 5 | 40 (29,53) | 62 | 0.003791355 | 0.219154444 | 11554 |
| Obsessive Compulsive Disorder | Psychiatric | Cluster 8 | 34 (27,44) | 34 | -0.019894832 | 0.816356484 | 12719 |
| Personality Disorder | Psychiatric | Cluster 8 | 34 (26,43) | 22 | -0.020128084 | 0.746858586 | 19293 |
| Schizophrenia Spectrum | Psychiatric | Cluster 7 | 37 (28,51) | 83 | 0.005144472 | 0.705293728 | 23651 |
| Substance Misuse | Psychiatric | Cluster 8 | 32 (25,43) | 21 | -0.013433911 | 0.960712717 | 49446 |
| Allergic/chronic Rhinitis | Respiratory | Cluster 5 | 40 (30,55) | 80 | 0.010817661 | 0.815641588 | 342301 |
| Asbestosis | Respiratory | Cluster 3 | 70 (63,78) | 78 | 0.129132785 | 0.957289562 | 3105 |
| Aspiration Pneumonitis | Respiratory | Cluster 2 | 79 (66,87) | 84 | 0.086659774 | 0.992724373 | 8297 |
| Asthma | Respiratory | Cluster 5 | 43 (31,58) | 84 | 0.016736077 | 0.969992547 | 274724 |
| Bronchiectasis | Respiratory | Cluster 3 | 66 (54,76) | 84 | 0.076728078 | 0.986947259 | 15426 |
| Chronic Obstructive Pulmonary Disease | Respiratory | Cluster 3 | 67 (58,76) | 84 | 0.109694926 | 0.991862342 | 107700 |
| Chronic Sinusitis | Respiratory | Cluster 5 | 44 (34,56) | 63 | 0.012187163 | 0.949719673 | 70312 |
| Hypertrophic Nasal Turbinates | Respiratory | Cluster 5 | 39 (30,50) | 50 | -0.011500469 | 0.847472021 | 10629 |
| Nasal Polyps | Respiratory | Cluster 5 | 48 (37,59.75) | 67 | 0.023720595 | 0.983299422 | 32943 |
| Pleural Effusion | Respiratory | Cluster 3 | 73 (60,83) | 84 | 0.084337848 | 0.998759356 | 40300 |
| Pleural Plaque | Respiratory | Cluster 3 | 73 (65,81) | 83 | 0.130003523 | 0.96929777 | 5350 |
| Pneumothorax | Respiratory | Cluster 4 | 40 (27,63) | 84 | 0.023363357 | 0.948613218 | 12765 |
| Pulmonary Collapse | Respiratory | Cluster 3 | 69 (56,80) | 84 | 0.080823324 | 0.995421577 | 14474 |
| Pulmonary Fibrosis | Respiratory | Cluster 3 | 74 (65,82) | 84 | 0.110997444 | 0.981793975 | 7153 |
| Respiratory Failure | Respiratory | Cluster 3 | 74 (62,83) | 84 | 0.095910695 | 0.995388369 | 20722 |
| Sleep apnoea | Respiratory | Cluster 5 | 52 (43,61) | 64 | 0.039823228 | 0.992494531 | 29506 |
| Acne | Skin | Cluster 9 | 29 (24,35) | 21 | -0.049870769 | 0.962700685 | 119445 |
| Actinic keratosis | Skin | Cluster 3 | 68 (59,76) | 83 | 0.126282758 | 0.998661991 | 78821 |
| Alopecia Areata | Skin | Cluster 5 | 36 (29,47) | 35 | -0.016720658 | 0.770087247 | 10271 |
| Dermatitis | Skin | Cluster 4 | 45 (33,60) | 82 | 0.025570539 | 0.990901444 | 522337 |
| Hidradenitis suppurativa | Skin | Cluster 6 | 36 (28,45) | 38 | -0.039010064 | 0.872990864 | 9301 |
| Lichen Planus | Skin | Cluster 4 | 54 (41,64) | 75 | 0.042407566 | 0.990078527 | 19741 |
| Pilonidal cyst/sinus | Skin | Cluster 9 | 29 (24,38) | 22 | -0.035472478 | 0.964286389 | 25529 |
| Psoriasis | Skin | Cluster 5 | 43 (32,58) | 72 | 0.015919414 | 0.937393133 | 99260 |
| Rosacea | Skin | Cluster 5 | 47 (37,59) | 68 | 0.02609977 | 0.979191995 | 78583 |
| Seborrheic Dermatitis | Skin | Cluster 4 | 46 (34,62) | 80 | 0.027238926 | 0.973239379 | 121253 |
| Urticaria | Skin | Cluster 5 | 43 (32,57) | 76 | 0.016755851 | 0.968568373 | 116459 |
| Vitiligo | Skin | Cluster 5 | 42 (31,54) | 71 | 0.006643746 | 0.692023942 | 9377 |

Supplementary Table S2. The cophenetic correlation coefficient (CCC) for different linkage methods using hierarchical agglomerative clustering.

| Linkage method | Average | Single | Complete | Ward |
| --- | --- | --- | --- | --- |
| CCC | 0.73253 | 0.49541 | 0.61314 | 0.59436 |

Supplementary Table S3. The optimal number of clusters (using the gap statistic) and the Dunn Index for different clustering algorithms. HAC = Hierarchical agglomerative clustering, PAM = partitioning around medioids.

| Algorithm | HAC (average linkage) | K-means | PAM | Spectral |
| --- | --- | --- | --- | --- |
| Optimal number of clusters | 18 | 9 | 18 | 10 |
| Dunn Index value | 0.12056 | 0.05584 | 0.06759 | 0.04806 |

Supplementary Table S4. The median age of first recorded diagnosis (25^th^ percentile, 75^th^ percentile) above the age of 20 years for 278 diseases in the 15 disease categories by age-related curve cluster.

|  | **Cardiovascular** | **Cancers** | **Respiratory** | **Eye** | **Musculoskeletal** | **Endocrine** | **Haematological or Immunological** | **Infections** | **Ear** | **Neurological** | **Genitourinary** | **Digestive** | **Benign Neoplasm** | **Skin** | **Psychiatric** |
| --- | --- | --- | --- | --- | --- | --- | --- | --- | --- | --- | --- | --- | --- | --- | --- |
| **Cluster 1** | 82 (82 ,82) |  |  |  | 80 (80 ,80) |  |  |  |  |  |  |  |  |  | 83 (83 ,83) |
| **Cluster 2** | 77 (75.75 ,77) | 77 (77 ,77) | 79 (79 ,79) | 75.5 (74.75 ,76.25) | 75 (75 ,75) | 79 (79 ,79) | 72 (72 ,72) | 73 (72 ,74) |  |  | 79 (79 ,79) |  |  |  |  |
| **Cluster 3** | 70 (67 ,72.5) | 70 (68 ,71) | 71.5 (68.5 ,73.25) | 69 (66.75 ,70.5) | 71 (67 ,71) | 67 (67 ,67) | 61 (60 ,65) | 64 (61.25 ,67.75) | 61 (61 ,61) | 67 (66 ,69.5) | 68.5 (64.75 ,71.75) | 67 (67 ,71) | 66 (66 ,66) | 68 (68 ,68) |  |
| **Cluster 4** | 59 (59 ,61) | 59 (59 ,61) | 40 (40 ,40) | 53.5 (47.75 ,60.25) | 58 (57 ,59.75) | 57 (56.5 ,57.5) | 56 (51.5 ,57) | 48 (47 ,53) | 55 (54.5 ,55.5) | 55.5 (48.25 ,56) | 57 (55 ,59) | 55 (54 ,57) | 59.5 (57.75 ,61.25) | 46 (45.5 ,50) |  |
| **Cluster 5** |  | 40 (39.5 ,40.5) | 43.5 (40.75 ,47) | 46 (45.5 ,46.5) | 45.5 (41 ,47) | 41 (38.5 ,43.5) | 40 (40 ,40) | 39 (38.25 ,39.75) |  | 41 (39.5 ,42.5) | 51 (48.5 ,53.5) | 42 (40 ,45.5) | 48 (43 ,50) | 43 (42 ,43) | 40 (40 ,40) |
| **Cluster 6** |  | 35 (35 ,35) |  |  |  |  |  | 36 (36 ,36) |  |  | 35 (35 ,35.5) |  | 32 (32 ,32) | 36 (36 ,36) |  |
| **Cluster 7** |  |  |  |  |  |  |  | 32.5 (32.25 ,32.75) |  |  |  |  |  |  | 37 (37 ,37) |
| **Cluster 8** |  |  |  |  |  |  |  |  |  | 32 (32 ,32) |  | 31 (31 ,31) |  |  | 34 (32 ,34) |
| **Cluster 9** |  |  |  |  |  |  |  |  |  |  |  |  |  | 29 (29 ,29) | 27 (27 ,27) |

**Supplementary Notes**

***Determining the optimal linkage method for hierarchical clustering***

In hierarchical clustering, the dissimilarity between two clusters can be measured using different linkage methods: single linkage (minimum Euclidean distance between diseases in the two clusters); complete linkage (maximum Euclidean distance between diseases in the two clusters); average linkage (average of all Euclidean distances between diseases in the two clusters); and Ward linkage (increase in the within cluster error sum-of-squares that results when two clusters are merged)^[[1]](#endnote-1),^^[[2]](#endnote-2)^. The cophenetic correlation coefficient measures the goodness-of-fit of the clustering algorithm by quantifying how well a dendrogram produced by a hierarchical clustering method corresponds to the original Euclidean distances. It is defined as the correlation of the matrix representing the height at which two diseases are merged in a dendrogram and the Euclidean distance matrix, with high values indicating a better fit^[[3]](#endnote-3),^^[[4]](#endnote-4)^. Using this approach, the average linkage method produced the highest cophenetic correlation coefficient (Supplementary Table S2).

***Using the gap statistic to determine the optimal number of clusters***

The gap statistic is a goodness-of-clustering measure that compares the log of the within-cluster sum of squared distances from the cluster mean (*log(W_k_*)) with its expectation under the null reference distribution of the data ($E_{n}^{*}$*log*($W_{k}^{*}$)) for each hypothetical number of clusters k. The null reference distribution is one with no obvious clustering that is generated by uniform sampling using Monte Carlo simulation. The gap function is defined as:

*Gap_n_(k) =* $E_{n}^{*}$*log*($W_{k}^{*})$- *log(W_k_*) (S1)

The optimal number of clusters is the smallest k such that

*Gap_n_(k) ≥ Gap_n_(k + 1) – s_k+1_* (S2)

where

*s_k_ =* $\sqrt{1+ \frac{1}{B}}$*sd(k)* (S3)

and *sd(k)* denotes the standard deviation of the *B* Monte Carlo replicates *log*($W_{k}^{*}$).

***The Dunn validation index***

The Dunn index is a metric for evaluating the performance of different clustering algorithms. It measures the ratio between the smallest distance between points in different clusters, and the largest distance within any of the clusters as follows^[[5]](#endnote-5)^:

*Dunn* = $\min_{1\leq i\leq k} \left\{ \min_{i+1\leq j\leq k} \left\{ \frac{\delta(c_{i}, c_{j} )}{\max_{1\leq l\leq k} \Delta(c_{l})} \right\} \right\}$ (S4)

where

$\delta(c_{i}, c_{j} )$ is the minimal distance between clusters *c_i_* and *c_j_*, and

$\Delta(c_{l})$ = $\max_{x,y \in c_{l}} \left\| x-y \right\|$ is the largest distance within a cluster *c_l_*, also known as the diameter of a cluster

A high value for the Dunn index indicates a compact and well-separated cluster.

1. Kaufman, L. and P.J. Rousseeuw, 1990. Finding Groups in Data: An Introduction to Cluster Analysis. Wiley, New York [↑](#endnote-ref-1)
2. Ward JHJ. Hierarchical Grouping to Optimize an Objective Function. Journal of the American Statistical Association. 1963. 58, 236–244 [↑](#endnote-ref-2)
3. Sokal RR, Rohlf FJ. The comparison of dendrograms by objective methods. Taxon 1962, 11: 33–40. 10.2307/1217208 [↑](#endnote-ref-3)
4. Saraçli S, Doğan N & Doğan İ. Comparison of hierarchical cluster analysis methods by cophenetic correlation. J Inequal Appl 2013, 203 (2013). https://doi.org/10.1186/1029-242X-2013-203 [↑](#endnote-ref-4)
5. Dunn JC. A fuzzy relative of the ISODATA process and its use in detecting compact well separated clusters. Cybernetics. 1973;3:32–57. 10.1080/01969727308546046 [↑](#endnote-ref-5)
